# Gut microbiota dysbiosis deteriorates immunoregulatory effects of tryptophan via colonic indole and LBP/HTR2B-mediated macrophage function

**DOI:** 10.1093/ismejo/wrae166

**Published:** 2024-08-24

**Authors:** Lili Jiang, Youling Hao, Dandan Han, Wenjian Dong, Aoyu Yang, Zhiyuan Sun, Yao Ge, Shuai Duan, Xiuwen Zhang, Zhaolai Dai

**Affiliations:** State Key Laboratory of Animal Nutrition and Feeding, College of Animal Science and Technology, China Agricultural University, Beijing 100193, P. R. China; State Key Laboratory of Animal Nutrition and Feeding, College of Animal Science and Technology, China Agricultural University, Beijing 100193, P. R. China; State Key Laboratory of Animal Nutrition and Feeding, College of Animal Science and Technology, China Agricultural University, Beijing 100193, P. R. China; State Key Laboratory of Animal Nutrition and Feeding, College of Animal Science and Technology, China Agricultural University, Beijing 100193, P. R. China; State Key Laboratory of Animal Nutrition and Feeding, College of Animal Science and Technology, China Agricultural University, Beijing 100193, P. R. China; State Key Laboratory of Animal Nutrition and Feeding, College of Animal Science and Technology, China Agricultural University, Beijing 100193, P. R. China; State Key Laboratory of Animal Nutrition and Feeding, College of Animal Science and Technology, China Agricultural University, Beijing 100193, P. R. China; State Key Laboratory of Animal Nutrition and Feeding, College of Animal Science and Technology, China Agricultural University, Beijing 100193, P. R. China; State Key Laboratory of Animal Nutrition and Feeding, College of Animal Science and Technology, China Agricultural University, Beijing 100193, P. R. China; State Key Laboratory of Animal Nutrition and Feeding, College of Animal Science and Technology, China Agricultural University, Beijing 100193, P. R. China

**Keywords:** tryptophan, antibiotics, indole, lipopolysaccharide-binding protein, HTR2B

## Abstract

Tryptophan (Trp) has been shown to regulate immune function by modulating gut serotonin (5-HT) metabolism and signaling. However, the mechanisms underlying the microbial modulation of gut 5-HT signaling in gut inflammation with gut microbiota dysbiosis require further investigation. Here, we investigated the effects of Trp supplementation on the composition and metabolism of the gut microbiome and 5-HT signaling-related gut immune function using a dextran sodium sulfate (DSS)-induced colitis mouse model coupled with antibiotic exposure. The results showed that antibiotic treatment before but not during DSS treatment decreased the immunoregulatory effects of Trp and aggravated gut inflammation and body weight loss in mice. Metagenomic analysis revealed that the fecal microbiota transplantation of Trp-enriched gut microbiota to recipient mice subject to antibiotic pre-exposure and DSS treatment alleviated inflammation by increasing the relative abundances of *Lactobacillus* and *Parabacteroides* and the microbial production of indole coupled with the activation of the 5-HT receptor 2B (HTR2B) in the colon. Transcriptomic analysis showed that HTR2B agonist administration strengthened the beneficial effects of Trp in DSS-induced colitis mice with antibiotic exposure by reducing gut lipopolysaccharide-binding protein (LBP) production, IκB-α/nuclear factor-κB signaling, and M1 macrophage polarization. Indole treatment reduced LBP production and M1 macrophage polarization both in mice with DSS-induced colitis and in lipopolysaccharide-treated mouse macrophages; however, the HTR2B antagonist reversed the effects of indole. Our findings provide the basis for developing new dietary and therapeutic interventions to improve gut microbiota dysbiosis-associated inflammatory gut disorders and diseases.

## Graphical Abstract

**Graphical Abstract:**
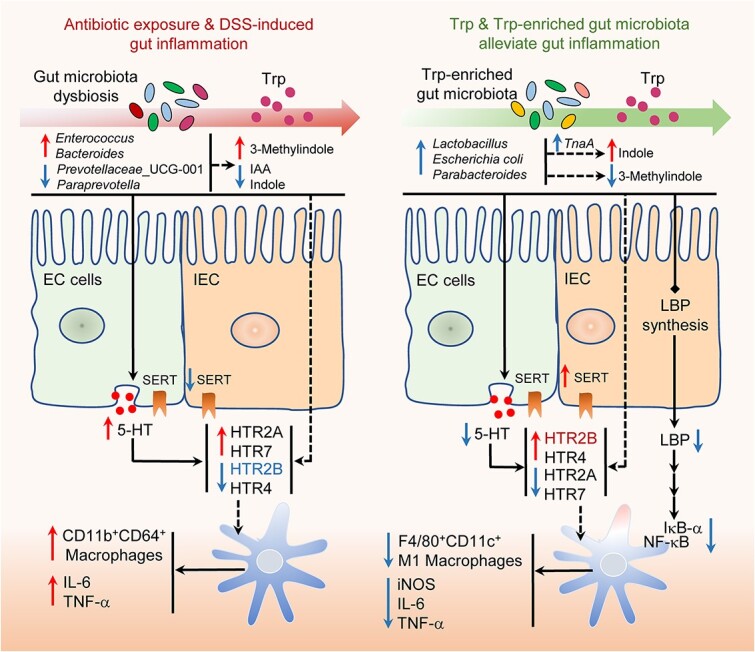

## Introduction

The homeostasis of the gut microecology is vital to maintain robust gut function [1]. Mounting evidence revealed that inflammatory bowel disease (IBD) and ulcerative colitis (UC) are closely linked to gut immune dysfunction and gut microbiota dysbiosis [1], which are regulated by dietary and therapeutic factors [2–4]. In IBD, the activation of gut immune cells such as T cells, natural killer (NK) cells, neutrophils, and macrophages and the production of inflammatory cytokines are observed [5]. Macrophage dysfunction in inflammation [6] contributes to the inappropriate activation of nuclear factor κB (NF-κB) signaling and nucleotide-binding oligomerization domain (NOD) leucine-rich repeat and pyrin domain-containing 3 (NLRP3) signaling [7]. Therefore, the regulation of macrophage polarization plays a critical role in gut inflammation.

Antibiotics (Abx) have been widely used for the treatment of gut inflammation. However, this practice has been questioned based on findings that Abx exposure further exacerbates gut microbiota dysbiosis in Crohn’s disease (CD) patients [8] and increases the numbers of gut *Escherichia coli* and the risk of IBD [9, 10]. Additionally, the gut microbiome and associated metabolites are implicated in the prevention and treatment of IBD and colorectal cancer (CRC) [11]; however, their interactions with Abx require further investigation.

Serotonin (5-HT) together with gut 5-HT receptors (HTRs) signaling has been shown to regulate gut inflammation [12, 13]. 5-HT modulates the gut microbiota and alters colitis susceptibility in tryptophan hydroxylase 1 (Tph1)^−/−^ mice [14]. In addition, 5-HT can activate HTR3A and induce NLRP3 inflammasome assembly, resulting in the release of IL-1β and CRC progression [15]. HTR4 knockout (KO) increased colitis in mice [16]. Mice with a tissue-specific KO of HTR2B in the intestine are more vulnerable to dextran sodium sulfate (DSS)-induced colitis [17]. Additionally, tryptophan (Trp) mitigated DSS-induced colitis in mice by upregulating *HTR1A* and *HTR4* expression and downregulating *HTR2A* expression in the colon [18]. Abx treatment eliminates the therapeutic effect of Trp on colitis in mice [19]. However, the detailed mechanisms of the immunoregulatory role of Trp via 5-HT signaling in gut microbiota dysbiosis-associated gut inflammation remain to be elucidated.

We hypothesize that the composition and metabolism of the gut microbiota and its coordination with gut 5-HT signaling are crucial for the immunoregulatory effects of Trp in the context of gut microbiota dysbiosis and inflammation. To test the above hypothesis, we combined Abx treatment, fecal microbiota transplantation (FMT), and HTR antagonist/agonist treatment to conduct a series of DSS-induced colitis mouse experiments. The findings from this study can provide the basis for the development of new dietary and therapeutic strategies to improve gut microbiota dysbiosis-related gut inflammation.

## Materials and methods

### Animals and experimental design

The six- to eight-week-old C57BL/6 male mice (19.0 ± 1.0 g) used in this study were purchased from Beijing HFK Bioscience (Beijing, China). Upon arrival, the mice were allowed to acclimate for 7 days before the experiment. The mice were housed in polycarbonate cages in a specific-pathogen-free environment and were kept at 22°C–25°C and 45%–55% relative humidity with a 12 h light/dark cycle. The mice had access to water and feed *ad libitum*. We used a standard rodent diet (Cat# 1032, Beijing HFK Bioscience) in this study as described previously [18]. All animal experimental procedures followed the guidelines of the Institutional Animal Care and Use Committee of China Agricultural University (Aw52101202-2-4, Aw11013202-1-1, Aw11013202-1-7).

### Tryptophan, antibiotic, and dextran sodium sulfate mouse experiment

A total of 50 mice were randomly divided into five groups: the control (Ctrl), DSS, Trp + DSS, Trp + Abx + DSS, and Trp + DSS + Abx groups. Each group had 10 mice. Colitis was induced by 2.5% (w/v) DSS in the water from Day 8 to Day 14. For Trp treatment, the mice were fed a 0.6 mg/mL Trp solution (providing 0.15 mg Trp per gram body weight per day) throughout the experiment [18]. For the Abx treatment, the mice were gavaged with 250 μL of Abx cocktail twice per day either before DSS treatment (Trp + Abx + DSS) or during DSS treatment (Trp + DSS + Abx) as previously described with modifications [19]. The mice in the other groups were gavaged with the same volume of phosphate-buffered saline (PBS). Body weight and feed intake were recorded daily. On Day 15, blood samples were collected and the mice were euthanized for sample collection and processing [18].

### Fecal microbiota transplantation mouse experiment

In total, 80 mice were used for the experiment. Before FMT, 40 recipient mice were treated with an Abx cocktail for 14 days as described previously [20]. At the same time, 40 donor mice were randomly divided into four groups: Ctrl, Trp, sterile fecal filtrate (SFF) of Trp, and Trp + Abx. The experimental procedures for the donor mice were the same as those in the first experiment. Fecal samples from the donor mice were collected daily and stored in 10% anaerobic glycerol at −80°C. On Day 15, the Abx-treated mice were randomly divided into four groups (FMT-Ctrl, FMT-Trp, FMT-SFF-Trp, and FMT-Trp + Abx) and transplanted with fecal microbiota from the corresponding donor mice. The FMT procedures were in accordance with the previous study [21]. SFF was prepared by centrifugation of the pooled fecal samples at 12 000 × g for 5 min to obtain the supernatant for subsequent gavage. The recipient mice were gavaged with 200 μL of the fecal microbiota/SFF for 17 days. On Day 11 after FMT, the recipient mice were treated with 2.5% DSS for 7 days. After DSS treatment, mice were euthanized for sampling.

### HTR2B agonist and HTR4 antagonist mouse experiment

HTR2B agonist (BW723C86) and HTR4 antagonist (GR113808) were selected to investigate the role of HTR2B and HTR4 in Abx -induced gut microbiota dysbiosis and inflammation. A total of 60 mice were randomly divided into six groups: Ctrl, DSS, Trp + DSS, Abx + Trp + DSS, Abx + Trp + DSS + BW723C86, and Abx + Trp + DSS+ BW723C86 + GR113808. The procedures used for the Abx and DSS treatments were the same as the FMT experiment. In parallel with DSS treatment, the mice were injected intraperitoneally with BW723C86 or GR113808 daily from Day 15 to Day 22 as described previously [16, 17, 22]. On Day 23, mice were euthanized for sampling.

### Indole and HTR2B antagonist/agonist mouse experiment

HTR2B antagonist (SB204741) and agonist (BW723C86) were used in this experiment to test whether HTR2B activation is essential for the immunoregulatory effect of indole. A total of 60 mice were randomly divided into six groups: Ctrl, Indole, DSS, Indole+DSS, Indole+DSS + SB204741, and Indole+DSS + BW723C86. The DSS and BW723C86 treatments were the same as those in the HTRs experiment described above. The dosage of SB204741 was the same as that in a previous study [17]. Indole was gavaged for 14 days as described before [23]. On Day 15, all the mice were euthanized for sampling.

### Statistical analysis

The data are expressed as the means ± SEMs. SPSS 22.0 (IBM, NY, USA) and Prism 9.0 (GraphPad, MA, USA) were used for statistical analysis. Differences between two groups were analyzed by *t*-test. Data were assessed by one-way analysis of variance followed by Tukey’s multiple comparison test. For the data that were not normally distributed, the Kruskal–Wallis H test was used to compare the differences. *P* < .05 was considered significantly different.

## Results

### Timing of antibiotic exposure impacts immunoregulatory effects of tryptophan on dextran sodium sulfate-induced colitis

**Figure 1:**
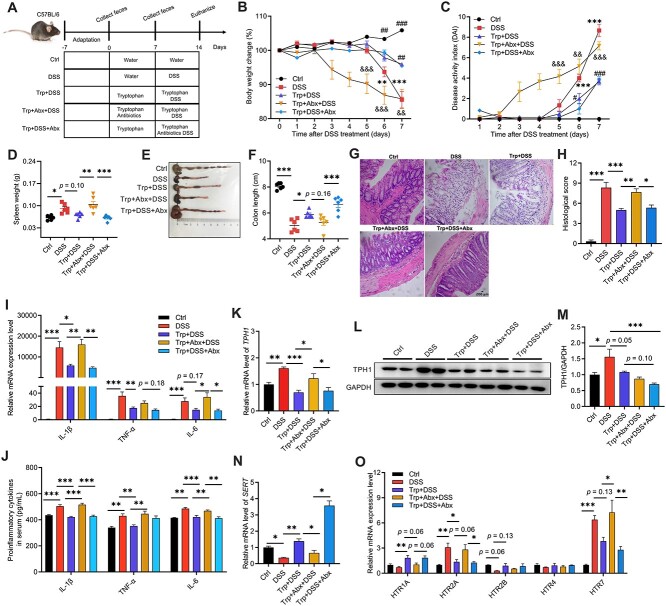
Effects of the timing of Abx exposure on the immunoregulatory effects of Trp and colonic 5-HT signaling in mice with DSS-induced colitis. (A) Schematic diagram of the experiment. (B) Body weight change. (C) Disease activity index scores. (D) Spleen weight. (E and F) Colon length. (G) H&E staining and (H) histological score of the colon. The mRNA and protein levels of inflammatory cytokines in the (I) colon and (J) serum of mice. The (K) mRNA levels and (L and M) protein abundances of TPH1 and the mRNA levels of (N) *SERT* and (O) *HTRs* in the colon. The data are the means ± SEMs. ^*^P < .05, ^*^^*^P < .01, and ^*^^*^^*^P < .001 compared to the Ctrl group; #*P* < .05, ##*P* < .01, and ###*P* < .001 compared to the DSS group; &*P* < .05, &&*P* < .01, and &&&*P* < .001 compared to the Trp + DSS group.

We first used a DSS-induced colitis mouse model to test the effects of the timing of Abx exposure on the colitis-alleviating effects of Trp (Fig. 1A). Trp administration alleviated DSS-induced body weight loss (Fig. 1B) and increase in disease activity index (DAI) score (Fig. 1C) relative to the DSS group. However, Abx pre-exposure (Trp + Abx + DSS) but not during DSS treatment (Trp + DSS + Abx) increased body weight loss (Fig. 1B) and DAI scores (Fig. 1C) on Day 5 and Day 6 after DSS treatment relative to the DSS group. Compared to the other groups, mice in the DSS and Trp + Abx + DSS groups had heavier spleens (Fig. 1D), shorter colons (Fig. 1E, 1F), increased colonic epithelial damage (Fig. 1G), and higher histological scores (Fig. 1H). The mRNA levels and concentrations of IL-1β, TNF-α, and IL-6 in the colon and serum were higher in the DSS and Trp + Abx + DSS groups than in the other groups (Fig. 1I and J).

Flow cytometric analysis of the abundances of immune cells in tissues revealed that Abx exposure before but not during DSS treatment had greater proportions of CD11b^+^/CD64^+^ macrophages in the colon, spleen, and plasma (Supplementary Fig. S1A), greater proportions of splenic CD11b^+^Ly6G^+^ neutrophils (Supplementary Fig. S1B), and greater proportions of CD11c^+^MHCII^+^ dendritic cells in the plasma (Supplementary Fig. S2). Further analysis of the subtypes of T helper (Th) cells and regulatory T (Treg) cells in the colon revealed that DSS treatment increased the proportions of CD4^+^IFN-γ^+^ Th1 cells, CD4^+^IL-4^+^ Th2 cells, and CD4^+^IL-17A^+^ Th17 cells relative to the Ctrl and Trp + DSS groups (Supplementary Fig. S3A). Abx exposure before but not during DSS treatment had greater proportions of Th1 and Th2 cells (Supplementary Fig. S3A). Mice in the DSS and Trp + DSS groups had greater proportions of Foxp3^+^ Treg cells relative to other groups (Supplementary Fig. S3B). There was no difference between the two Abx treatment groups in the proportions of CD3^+^ T cells, Foxp3^+^ Treg cells, B cells, or NK cells in tissues (Supplementary Figs S2A and B, S3B and S4).

### Antibiotic pre-exposure regulates colonic tryptophan metabolism and serotonin receptors in tryptophan-treated mice

We further analyzed the mRNA and protein levels of tryptophan hydroxylase 1 (TPH1), 5-HT reuptake transporter (SERT), and HTRs in the colon. Mice in the DSS group had higher mRNA and protein levels of TPH1 but lower levels of *SERT* than the other groups (Fig. 1K, L, and M). Abx pre-exposure had higher mRNA levels of *TPH1*, *HTR1A*, and *HTR7* but lower mRNA levels of *SERT*, *HTR1A*, and *HTR2B* relative to the Trp + DSS and Trp + DSS + Abx groups (Fig. 1K, N, and O).

Metabolomics combined with HPLC analysis of the Trp metabolites in the colon revealed that DSS treatment reduced the levels of kynurenic acid, xanthurenic acid, nicotinic acid, indole-3-lactic acid (ILA), indole-3-acetic acid (IAA), indole-3-propionic acid (IPA), and indole-3-carboxaldehyde in the colonic luminal content (Supplementary Table S1). Trp + DSS treated mice had higher levels of kynurenic acid, ILA, IPA, IAA, indole, and 3-methylindole in the colonic lumen than the DSS group (Supplementary Table S1; Supplementary Fig. S5C and D). Abx exposure, regardless of timing, resulted in increased levels of ILA in the colonic lumen (Supplementary Table S1). Additionally, the mice in the DSS and Trp + Abx + DSS groups had higher levels of 5-HT in the colon relative to the Trp + DSS group (Supplementary Fig. S5A).

### Effects of tryptophan treatment and antibiotic exposure on the fecal microbiota of mice with dextran sodium sulfate-induced colitis

**Figure 2:**
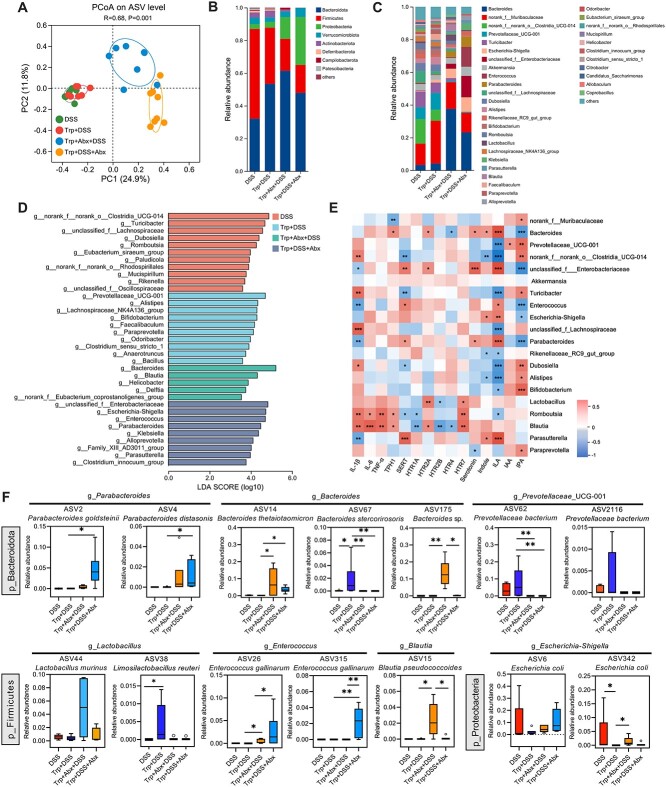
Tryptophan treatment and the timing of Abx exposure differentially regulate the gut microbiota of mice with DSS-induced colitis. (A) Principal coordinate analysis of the colonic microbiota of mice based on the Bray–Curtis distance. The relative abundance at the (B) phylum level and (C) genus level. (D) LEfSe analysis of differentially-enriched bacteria at the genus level. (E) Spearman correlation analysis between microbiota and cytokines, 5-HT metabolism, HTRs, and indole metabolites. (F) Box plot comparison of the top 20 differential ASVs among the treatment groups. ^*^P < .05, ^*^^*^P < .01, and ^*^^*^^*^P < .001.

We employed bacterial 16S rRNA gene sequencing analysis to identify the differences in the effects of Trp supplementation and the timing of Abx exposure on fecal microbiota composition. The results revealed that Abx exposure both before and during DSS treatment reduced α-diversity relative to the DSS and Trp + DSS groups (Supplementary Table S2). Principal coordinate analysis (PCoA) revealed that the timing of Abx exposure had different effects on microbiota composition relative to the DSS and Trp + DSS groups (Fig. 2A) with reduced relative abundances of *Firmicutes* and *Prevotella* but increased relative abundances of *Proteobacteria*, *Bacteroides*, and *Escherichia*-*Shigella* (Fig. 2B and C). The mice treated with Abx had the same enterotype as those in the DSS and Trp + DSS groups (Supplementary Fig. S6C). Linear discriminant analysis Effect Size (LEfSe) analysis at the genus level coupled with box plot comparisons of the top 20 differential ASVs revealed that Trp + DSS treatment had greater relative abundances of *Prevotellaceae*-UCG-001, *Allistipes*, *Bifidobacterium*, *Paraprevotella*, *Bacteroides stercorirosoris* (ASV67), and *Limosilactobacillus reuteri* (ASV38) and lower relative abundances of *E. coli* (ASV342) relative to the DSS group (Fig. 2D, 2F). The relative abundances of *Parabacteroidetes distasonis* (ASV4), *Bacteroides thetaiotaomicron* (ASV14), and *Enterococcus gallinarum* (ASV26) were greater in the Abx-exposed groups than in the DSS and Trp + DSS groups (Fig. 2F). However, Abx pre-exposure increased the relative abundances of *Helicobacter*, *Bacteroides* sp. (ASV175), *Blautia pseudococcoides* (ASV15), and *E. coli* (ASV342) relative to the Trp + DSS and Trp + DSS + Abx groups (Fig. 2D and F). The relative abundances of *Klebsiella, Alloprevotella, Parasutterella*, *Parabacteroidetes goldsteinii* (ASV2), and *E. gallinarum* (ASV26, ASV315) were greater in mice of the Trp + DSS + Abx group relative to other DSS treatment groups (Fig. 2D, 2F).

**Figure 3:**
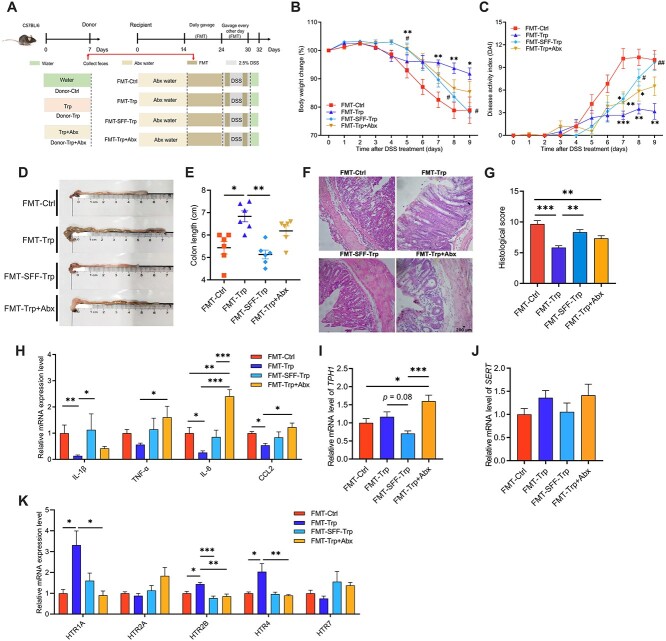
FMT of the Trp-enriched microbiota to Abx-treated mice mitigates DSS-induced colitis. (A) Schematic diagram of the FMT experiment. (B) Body weight change. (C) Disease activity index scores. (D and E) Colon length. (F) H&E staining and (G) histological score of the colon. The mRNA levels of (H) inflammatory cytokines, (I) *TPH1*, (J) *SERT*, and (K) *HTRs* in the colon. The data are the means ± SEMs. ^*^P < .05, ^*^^*^P < .01, and ^*^^*^^*^P < .001 compared to the FMT-Ctrl group; #*P* < .05, ##*P* < .01, and ###*P* < .001 compare to the FMT-Trp group.

We performed Spearman’s correlation analysis to elucidate the associations between the gut microbiota and inflammatory cytokines, HTRs, and Trp metabolites. The results showed that *Romboutsia* and *Blautia* were positively correlated with the levels of pro-inflammatory cytokines (*IL*-*1β*, *IL-6*, and *TNF-α*), *HTR2A*, *HTR7*, and *TPH1* but negatively correlated with the levels of *HTR2B* and *HTR4* (Fig. 2E). Additionally, the abundances of *Parabacteroides* and *Parasutterella* were positively correlated with *SERT* but negatively correlated with *IL*-*1β* (Fig. 2E). *Bacteroides*, *Parabacteroides*, *Parasutterella*, and *Escherichia*-*Shigella* were positively correlated with indole and ILA but negatively correlated with IPA (Fig. 2E).

### Fecal microbiota transplantation of tryptophan-enriched microbiota to antibiotic pre-exposed mice alleviates dextran sodium sulfate-induced colitis by regulating serotonin receptors and indole metabolism

To elucidate the effects of the Trp-enriched gut microbiota on the regulation of the colonic immune response in inflammation, we pretreated the mice with Abx for 14 days to induce gut microbiota dysbiosis and then subjected them to FMT followed by DSS treatment (Fig. 3A). After DSS treatment, mice FMT with microbiota from the Trp-pretreated mice (FMT-Trp) had lower body weight loss, DAI and colonic histological scores but longer colon than mice FMT with microbiota from the Ctrl (FMT-Ctrl), the SFF of FMT-Trp (FMT-SFF-Trp) group, and the Trp- and Abx-pretreatment (FMT-Trp + Abx) group (Fig. 3B–G). Compared with other groups, the mice in the FMT-Trp group had lower levels of *IL-1β, IL-6*, and *CCL2* but higher levels of *HTR1A*, *HTR2B*, and *HTR4* (Fig. 3H and K). In addition, *TPH1* mRNA levels were higher in the FMT-Trp + Abx group than in the other groups (Fig. 3I).

Analysis of Trp metabolites revealed that mice in the FMT-Trp + Abx group had higher levels of 5-HT in the colon and serum than did mice in the FMT-Trp and FMT-SFF-Trp groups (Supplementary Fig. S7A). Additionally, mice in the FMT-Trp group had higher levels of indole, but lower levels of 3-methylindole in the colon than did those in the FMT-Ctrl and FMT-Trp + Abx groups (Supplementary Fig. S7C and D).

### Fecal microbiota transplantation of tryptophan-enriched microbiota to antibiotic pre-exposed mice regulates the structure and indole metabolism of the colonic microbiome

We employed bacterial 16S rRNA gene sequencing analysis of the fecal and colonic microbiota to elucidate the composition of the Trp-enriched microbiota and its effects on the colonic microbiome of the recipient mice. For the FMT donor mice, PCoA analysis showed that Trp treatment and Abx exposure affected the fecal microbiota composition compared to the Ctrl group (Supplementary Fig. S8A). Compared with the Ctrl or Trp + Abx groups, the Trp group had greater relative abundances of *Firmicutes*, *Lactobacillus*, and *Desulfovibrio* (Supplementary Fig. S8B–E). Mice treated with Abx (Trp + Abx) had greater abundances of fecal *Proteobacteria* and *Enterococcus* relative to the other groups (Supplementary Fig. S8B and E). For the recipient mice, PCoA analysis showed that the fecal microbiota compositions differed before and after 14 days of Abx treatment (Supplementary Fig. S9A), with increased α-diversity after Abx treatment (Supplementary Table S3). Abx treatment reduced the abundance of *Firmicutes*, *Lactobacillus*, *Prevotellaceae*_UCG-001, *Alistipes*, *Desulfovibrio*, and *Bacteroides* but increased the abundance of *Proteobacteria* and *Staphylococcus* (Supplementary Fig. S9B–D).

After FMT and DSS treatment, there was no difference in the α-diversity of the colonic microbiota of the mice FMT with Trp-enriched microbiota or SFF from the Trp treatment groups (Supplementary Table S4). PCoA analysis revealed a clear separation of the colonic microbiota composition of the recipient mice among the different treatment groups (Supplementary Fig. S10A). Compared to the FMT-Ctrl group, mice FMT with microbiota from the Trp treatment groups had greater relative abundances of *Proteobacteria*, *E. coli* (ASV6), and *Parabacteroides distasonis* (ASV4; Supplementary Fig. S10B, C, and F). Mice in the FMT-Trp group had greater relative abundances of *Lactobacillus* [e.g. *L. murinus* (ASV44) and *Lactobacillus johnsonii* (ASV42)], *Parabacteroides goldsteinii* (ASV2), *Enterococcus faecium* (ASV561), and *Prevotellaceae*_UCG-001 relative to the FMT-Trp + Abx groups (Supplementary Fig. S10B, C, D, and F). However, the relative abundance of *E. coli* (ASV342) was greater in the FMT-Trp + Abx group than in the other groups (Supplementary Fig. S10F).

Correlation analysis revealed that the abundances of *Parabacteroides* and *Lactobacillus* were negatively correlated with the levels of pro-inflammatory cytokines but were positively correlated with the levels of anti-inflammatory HTR2B and HTR4 and colonic indole (Supplementary Fig. S10E). However, the abundance of *Alloprevotella* was positively correlated with the levels of proinflammatory cytokines but negatively correlated with HTR1A, HTR4, and colonic indole (Supplementary Fig. S10E). Additionally, the abundance of *Klebsiella* was negatively correlated with *IL-1β* but positively correlated with indole concentration in the colon (Supplementary Fig. S10E).

### Indole production was upregulated in the colonic microbiome of mice after fecal microbiota transplantation of tryptophan-enriched microbiota

**Figure 4:**
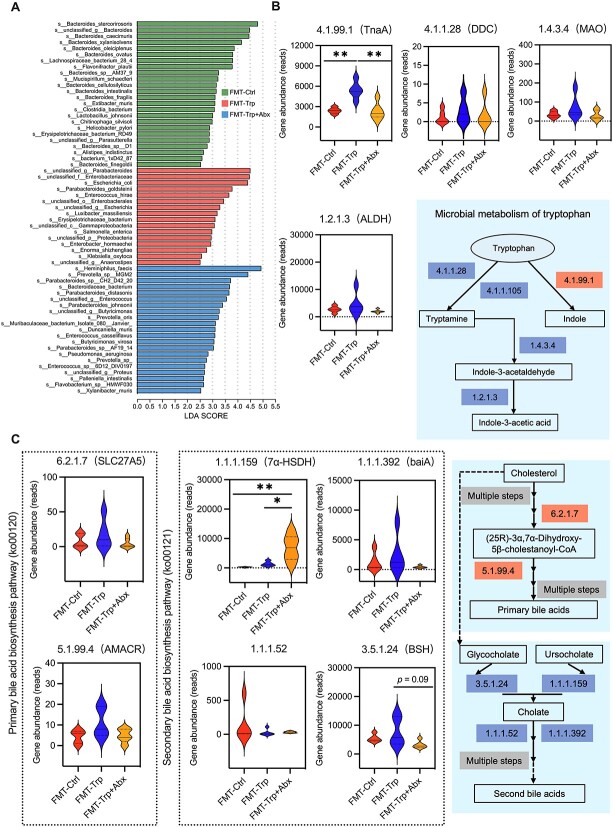
Metagenomic analysis of the effects of FMT on the gut microbiota metabolism of recipient mice. (A) LEfSe analysis of differentially-enriched gut microbiota at the species level. KEGG pathway analysis of the gene abundance of the enzymes involved in the microbial metabolism of (B) tryptophan and (C) bile acids. ^*^P < .05, ^*^^*^P < .01.

We employed metagenomic sequencing analysis to further identify the differences in the composition and microbial metabolism of the colonic microbiome of the recipient mice. For the fecal microbiota composition of the donor mice, the relative abundances of *Bacteroides cellulosilyticus* and *Lactobacillus* were greater in the Trp group (Supplementary Fig. S11A). After FMT, the colonic microbiota of the mice in the FMT-Trp group had greater relative abundances of *Parabacteroides* and *Enterococcus*, which is similar to the 16S rRNA gene sequencing results (Fig. 4A).

Microbial gene catalog analysis showed functional separation between the Ctrl group and treatment groups of the donor mice and recipient mice (Supplementary Fig. S11B and C). In donor mice, aminoacyl-tRNA and peptidoglycan biosynthesis and purine metabolism were enriched in the Trp-enriched fecal microbiota (Supplementary Fig. S11D). For the recipient mice, pathways involved in microbial metabolism in diverse environments, carbohydrate uptake, and propanoate metabolism were enriched in the FMT-Trp group (Supplementary Fig. S11E). Additionally, the colonic microbiota of the FMT-Trp + Abx group was enriched in 16 pathways, including lipopolysaccharide biosynthesis (Supplementary Fig. S11E). We further analyzed the differentially-enriched enzymes in the microbial metabolism pathways of Trp and bile acids. The results showed that tryptophanase (TnaA), a key enzyme involved in indole production, was enriched in the colonic microbiome of the mice in the FMT-Trp group relative to the other groups (Fig. 4B). Additionally, bile acid hydrolase was enriched in the FMT-Trp group relative to the FMT-Trp + Abx group (Fig. 4C).

### Activation of HTR2B alleviates dextran sodium sulfate-induced colitis by reducing M1 macrophage polarization in mice pretreated with antibiotics and tryptophan

**Figure 5:**
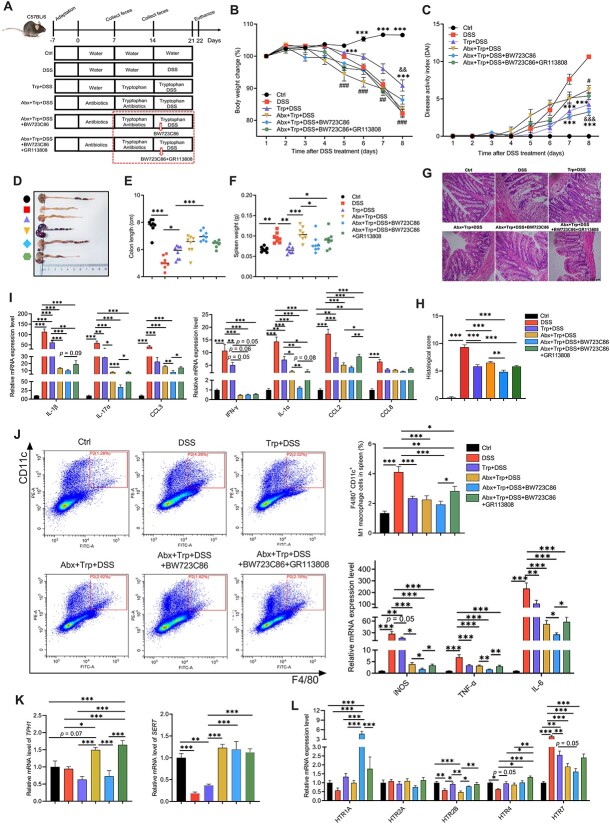
Activation of HTR2B ameliorates DSS-induced colitis by regulating of M1 macrophage polarization and 5-HT homeostasis in the colon. (A) Schematic diagram of the experiment. (B) Body weight change. (C) Disease activity index scores. (D and E) Colon length. (F) Spleen weight. (G) H&E staining and (H) histological score of the colon. (I) Levels of inflammatory cytokines. (J) Flow cytometry of M1 macrophages and the expression of marker genes. Expression of (K) *TPH1* and *SERT*, and (L) *HTRs*. The data are the means ± SEMs. ^*^P < .05, ^*^^*^P < .01, and ^*^^*^^*^P < .001 compared to the DSS group; #*P* < .05, ##*P* < .01, and ###*P* < .001 compared to the Trp + DSS group; &*P* < .05, &&*P* < .01, and &&&*P* < .001 compared to the Abx + Trp + DSS group.

We combined the use of an HTR2B agonist (BW723C86) and an HTR4 antagonist (GR113808) to elucidate the mechanism by which HTRs affect the immunoregulatory effects of Trp in mice with Abx pre-exposure and DSS-induced colitis (Fig. 5A). Mice treated with the HTR2B agonist (Abx + Trp + DSS + BW723C86) had a lower percentage of body weight loss and DAI score on Day 8 after DSS treatment relative to the DSS and Abx exposure (Abx + Trp + DSS) groups (Fig. 5B and C). However, co-treatment with the HTR4 antagonist and HTR2B agonist (Abx + Trp + DSS + BW723C86+ GR113808) reversed these effects (Fig. 5B and C). Compared with the Abx-exposed mice, the mice treated with the HTR2B agonist had longer colons, lighter spleens and less morphological damage (Fig. 5D–H). Compared to Abx-exposed mice, mice treated with the HTR2B agonist alone rather than co-treated with the HTR2B agonist and HTR4 antagonist had lower mRNA levels of *IL-1α*, *IL-17α*, and *CCL3* (Fig. 5I).

Analysis of the proportions of immune cells in the spleen showed that mice in the Trp + DSS and Abx-treatment groups had lower proportions of F4/80^+^CD11c^+^ M1 macrophages relative to the DSS group (Fig. 5J). M1 macrophages were more abundant in mice in the HTR4 antagonist treatment group than those in the other Trp-treatment groups (Fig. 5J). Similar effects of HTR agonist/antagonist treatment on the proportions of F4/80^+^CD206^+^ M2 macrophages were observed (Supplementary Fig. S12A). Analysis of the expression of macrophage polarization markers revealed that mice in the HTR2B agonist group had lower mRNA levels of the M1 marker genes *IL-6*, *TNF-α*, and *iNOS* and the M2 marker gene *Arg-1* relative to the Abx exposure group and the HTR2B agonist and HTR4 antagonist co-treatment group (Fig. 5J; Supplementary Fig. S12A). Similar observations were made for splenic CD11b^+^Ly6G^+^ neutrophils (Supplementary Fig. S12B).

Analysis of 5-HT metabolism and signaling in the colon showed that mice in the HTR2B agonist group had lower levels of *TPH1* but higher levels of *HTR1A* and *HTR2B* relative to the Abx exposure group and the HTR2B agonist and HTR4 antagonist co-treatment group (Fig. 5K and L).

### HTR2B agonist inhibits LBP production and IκB-α/NF-κB signaling and improves the immunoregulatory effects of tryptophan in antibiotic-treated mice

**Figure 6:**
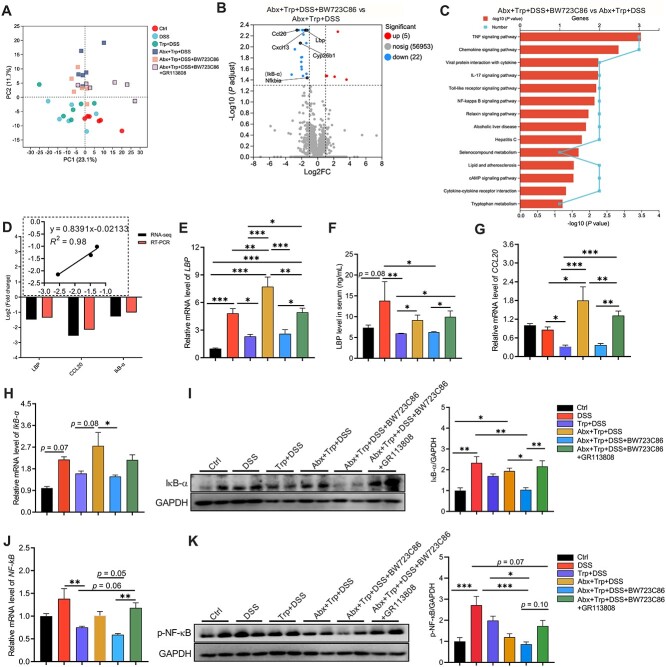
Activation of HTR2B improves the immunoregulatory effects of Trp in Abx-treated mice by inhibiting LBP production and IκB-α/NF-κB signaling. (A) PCA plot of the transcriptional profiles of the treatment groups. (B) Volcano plot of differentially-expressed genes between groups. (C) KEGG analysis of the differentially-enriched pathways between groups. (D) Fold change of differential genes in RNA-seq analysis and their correlation with gene expression. Expression and levels of LBP in the (E) colon and (F) serum. Expression of (G) *CCL20*, (H) *IκB-α*, and (J) *NF-κB*, and the protein abundance of (I) IκB-α and (K) p-NF-κB. The data are the means ± SEMs. ^*^P < .05, ^*^^*^P < .01, ^*^^*^^*^P < .001.

We further employed transcriptomic analysis of the colon to elucidate the mechanism by which HTRs affect the immunoregulatory effects of Trp in mice with Abx pre-exposure and DSS-induced colitis. PCoA analysis showed a clear separation of the transcriptomic profiles in the colon of the mice among the treatment groups (Fig. 6A). Differential genes and related annotated pathways were identified (Supplementary Fig. S13A–C; Fig. 6B). Compared to the mice in the Abx + Trp + DSS group, those in the HTR2B agonist treatment group exhibited five upregulated genes and 22 downregulated genes, including *LBP*, *CCL20*, and *IκB-α* (Fig. 6B). KEGG enrichment analysis of the DEGs revealed that the NF-κB, TNF*-α*, and IL-17 signaling pathways were enriched (Fig. 6C; Supplementary Fig. S13D and E).

The differentially-expressed genes were further validated by real-time-polymerase chain reaction (PCR), Western-blotting and ELISA. Correlation analysis revealed that the real-time PCR data were comparable to the RNA-seq data (Fig. 6D). Mice in the HTR2B agonist treatment group had lower mRNA levels of *LBP*, *CCL20*, *IκB-α*, and *NF-κB* in the colon and lower serum levels of LBP relative to the Abx exposure group and the HTR2B agonist and HTR4 antagonist co-treatment group (Fig. 6E–H and 6J). Similar effects of the HTR2B agonist on the protein abundance of IκB-α and p-NF-κB in the colon were observed (Fig. 6I and K).

### Indole upregulates *HTR2B* and reduces LBP and M1 macrophage polarization in dextran sodium sulfate-treated mice

**Figure 7:**
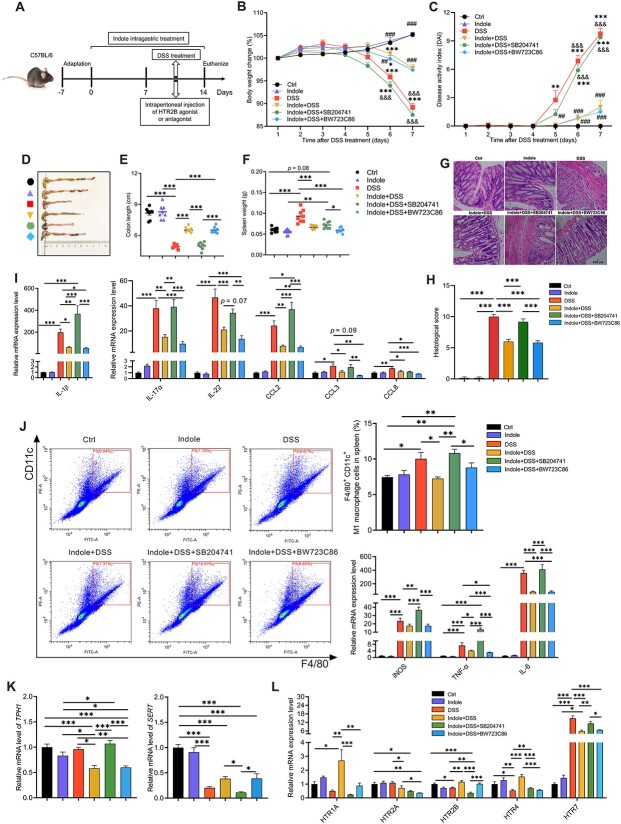
Indole upregulates HTR2B and reduces LBP production and M1 macrophage polarization in mice with DSS-induced colitis. (A) Schematic diagram of the experiment. (B) Body weight change. (C) Disease activity index scores. (D and E) Colon length. (F) Spleen weight. (G) H&E staining and (H) histological score of the colon. (I) Expression of inflammatory cytokines in the colon. (J) Flow cytometry analysis of M1 macrophages and the expression of marker genes. Expression of (K) *TPH1*and *SERT*, and (L) *HTRs* in the colon. The data are the means ± SEMs. ^*^P < .05, ^*^^*^P < .01, and ^*^^*^^*^P < .001 compared to the Ctrl group; #*P* < .05, ##*P* < .01, and ###*P* < .001 compared to the DSS group; &*P* < .05, &&*P* < .01, and &&&*P* < .001 compared to the indole + DSS group.

To validate the mechanisms of HTR2B in the regulation of body LBP production and macrophage behavior via indole in colonic inflammation, we treated mice with HTR2B antagonist (SB204741) or agonist (BW723C86) together with indole and DSS (Fig. 7A). We found that mice in the DSS and HTR2B antagonist treatment groups had higher percentages of body weight loss and higher DAI scores on Day 6 and Day 7 after DSS treatment relative to the Indole + DSS and HTR2B agonist treatment groups (Fig. 7B, 7C). Similarly, when compared with those in the Indole + DSS and HTR2B agonist treatment groups, the mice in the DSS and HTR2B antagonist treatment (Indole + DSS + SB204741) groups had shorter colons, heavier spleens, and higher histological scores (Fig. 7D–H). Similar effects of HTR2B antagonist treatment were found for the expression of *LBP*, pro-inflammatory cytokines (*IL-1β*, *IL-17α*, and *IL-22*), chemokines (*CCL2* and *CCL3*), M1 macrophage polarization markers (*IL-6*, *TNF-α*, and *iNOS*), and M2 macrophage polarization markers (*Arg-1* and *IL-10*) in the colon (Fig. 7I and J; Supplementary Figs S14A and S15A).

Analysis of genes related to 5-HT metabolism and signaling revealed that the DSS and HTR2B antagonist treatment groups had higher mRNA levels of *TPH1* and *HTR7* but lower mRNA levels of *SERT*, *HTR1A*, and *HTR2B* than the Indole + DSS and HTR2B agonist treatment groups (Fig. 7K and L).

Further analysis of the differentiation and function of macrophages showed that the mice in the DSS and HTR2B antagonist treatment groups had higher percentages of splenic F4/80^+^CD11c^+^ M1 macrophages and F4/80^+^CD206^+^ M2 macrophages relative to the Indole + DSS group (Fig. 7J; Supplementary Fig. S15A). We confirmed the above findings by treating mouse RAW264.7 macrophages with an HTR2B antagonist (SB204741) in an LPS-induced inflammation model (Supplementary Fig. S16A). Compared with those in the LPS group, the RAW264.7 cells pretreated with indole before LPS (Indole + LPS) had lower mRNA levels of *LBP*, *IL-1β*, M1 macrophage polarization markers (*IL-6* and *TNF-α*), *IκB-α*, and *NF-κB* (Supplementary Fig. S16). However, treatment with an HTR2B antagonist before LPS treatment (Indole+SB204741 + LPS) reversed the abovementioned effects of indole (Supplementary Fig. S16).

## Discussion

Gut microbiota dysbiosis induced by inflammation and Abx treatment is an important factor that facilitates the gut immune stimulation and worsens inflammatory gut disorders and diseases [24]. Mounting evidence suggests that the microbial metabolism of dietary and host-derived compounds, especially Trp, plays a crucial role in modulating the gut immune response [12, 25]. In this study, we focused on the microbial modulation of the gut 5-HT pathway, regulated by Trp, in gut microbiota dysbiosis and inflammation. The major findings of the present study are as follows: (i) Abx exposure before but not during DSS treatment deteriorates the immunoregulatory effects of Trp; (ii) Trp-enriched gut microbiota and microbial indole are essential for alleviating DSS-induced colonic inflammation after Abx -induced gut microbiota dysbiosis; (iii) the activation of HTR2B and the reduction of LBP by indole inhibit M1 macrophage polarization in colonic inflammation.

The robustness of the gut microbiome and the timing of Abx treatment are two important factors in dietary and therapeutic interventions for gut inflammation. Antibiotics are a double-edged sword with significant efficacy in the treatment of gut inflammation. However, they cause gut microbiota dysbiosis, Abx resistance, and chronic inflammation at the same time [26]. It was demonstrated that the effect of a Trp-enriched diet on the DSS-induced colitis mice was obstructed by Abx exposure [19]. However, the microbiota composition and metabolism responsible for dietary Trp and Abx treatment are not clear. Trp was shown to increase the abundance of *Lactobacillus* in the intestine of weaning piglets [27]. *P. goldsteinii* was shown to protect against gut inflammation and related diseases [28]. In addition, *Prevotellaceae*_UCG-001 was observed to be associated with immunoregulatory effects via the production of butyrate [29]. Our present study showed that in Abx-induced gut microbiota dysbiosis, the relative abundance of *Prevotellaceae*_UCG-001 decreased (Supplementary Fig. S9C). Trp supplementation before DSS treatment or FMT of the Trp-enriched microbiota increased the relative abundance of *Prevotellaceae*_UCG-001 (Fig. 2F; Supplementary Fig. S10C). Additionally, only under the conditions of Abx and Trp co-treatment or Trp supplementation after Abx exposure did the abundance of *Enterococcus* increase (Fig. 2F; Supplementary Fig. S10F). Although Trp supplementation increased the relative abundance of *Lactobacillus* in gut microbiota dysbiosis, their responses to Trp supplementation were DSS- or Abx-dependent. This was evidenced by increased abundances of *L. reuteri* (ASV38) after DSS treatment and *Lactobacillus murinus* (ASV44) after Abx treatment (Fig. 2F; Supplementary Fig. S10F). Studies have revealed that *Pevotella* species are sensitive to many Abx and bile acids [30, 31]. We suspect that bacteria belonging to *Prevotellaceae*_UCG-001 are more susceptible to Abx treatment and the Trp-altered colonic bile repertoire (Fig. 4) than *Lactobacillus* and *Enterococcus*, the abundances of which decrease after Trp and Abx administration (Fig. 2F). Further studies are warranted to uncover their immunoregulatory role in gut microbiota dysbiosis [32].

Microbial mediation of gut HTR signaling, regulated by Trp, is crucial for tuning gut epithelial function and inflammation. In gut inflammation, increased intestinal permeability was found to be accompanied by increased serum LBP, immune cell infiltration and signaling [33]. Trp or *Lactobacillus* reversed the DSS-induced upregulation of *HTR2A* and *HTR7* expression and downregulation of *HTR2B* and *HTR4* expression in the colon [18, 34]. Additionally, *Lactobacillus* reduces DSS-induced 5-HT production and accumulation in the colon [18, 35]. To date, the regulatory role of Trp in gut HTR2B signaling is not clear. In hepatocytes, the activation of HTR2B maintains blood glucose levels by promoting gluconeogenesis and preventing glucose uptake [36]. In liver Kupffer cells, HTR2B is preferentially expressed by the M2 macrophages [37]. However, the KO of HTR2B in the intestinal epithelial cells of mice aggravates the severity of AOM/DSS-induced colitis by inhibiting TGF-β/SMAD signaling and activating IL-6/STAT3 signaling [17]. To date, the microbiota-mediated regulation of gut HTRs is not yet clear. *Lactobacillus rhamnosus* GG was shown to upregulate gut *HTR4* expression and increase the abundance of *Alistipes* and *Allobaculum* in mice [38]. Further studies are warranted to uncover the mechanisms of the Trp-enriched microbiota in gut inflammation via HTR2B.

The findings of the present study may have important translational applications in the intervention and treatment of inflammatory gut disorders and diseases. In CD patients, the abundances of *Enterobacteriaceae* and *Fusobacteriaceae* increased but the abundances of *Erysipelotrichales*, *Bacteroidales*, and *Clostridiales* decreased [8]. Abx use in patients amplifies the microbial dysbiosis associated with CD [8]. Additionally, early-life Abx exposure increases the percentage of children who develop IBD [39]. This suggests the importance of the timing of Abx exposure on the progression and outcome of IBD. In addition, an increase in *TPH1* expression and a reduction in *SERT* expression were found in the inflamed colon of IBD patients, accompanied by increased levels of 5-HT and reduced levels of microbial indole metabolites in the blood [14, 40]. Therefore, FMT has been studied as a potential therapy for treating gut immune-related diseases such as IBD and type 1 diabetes to restore a healthy and functional gut microbiome [41, 42]. In the present study, similar changes in microbiota composition and metabolism were found in the colon after DSS or Abx treatment. Our current findings suggest that FMT using the microbiota from healthy donors may not be sufficient to restore the healthy gut microbiota in IBD patients with Abx exposure. Potential translational aspects and strategies for FMT-related therapy for these patients include (i) tuning the donor gut microbiota with dietary interventions such as probiotics and Trp-enriched diets to enrich specific microbial communities (e.g. *Prevotella* and *Faecalibacterium*) and promote the production of microbial metabolites (e.g. indole, IAA, IPA) before FMT and (ii) administering proper dosages of HTR2B and HTR4 agonists and indole metabolites together with FMT to facilitate the anti-inflammatory and healing processes. However, it is important to understand the baseline microbiome of patients and wisely select probiotic bacteria and metabolites [43, 44].

## Conclusions

Abx treatment before but not during DSS treatment deteriorates the immunoregulatory effects of Trp in DSS-induced colitis in mice. Trp-enriched gut microbiota and microbial indole are essential for the alleviation of DSS-induced colonic inflammation with gut microbiota dysbiosis. The reduction of LBP and the activation of HTR2B via indole reduce M1 macrophage polarization during colonic inflammation. The findings of our study provide the basis for applying the use of Trp-enriched gut microbiota and metabolites in the intervention and treatment of gut microbiota dysbiosis-associated inflammatory gut disorders and diseases in humans and animals (Supplementary Fig. S17).

## Supplementary Material

Supplemental_Materials_wrae166

## Data Availability

The data is available within the article and its supplementary materials. The datasets supporting the conclusions of this article are available in the NCBI Sequence Read Archive (SRA) repository under accession number PRJNA1030954, PRJNA1031700 and PRJNA1032707.
