# Supplementary material for: Gut microbiota dysbiosis deteriorates immunoregulatory effects of tryptophan via colonic indole and LBP/HTR2B-mediated macrophage function
Source: ISME J. 2024 Aug 24;18(1):wrae166. doi: 10.1093/ismejo/wrae166 (PMC11421149; doi:10.1093/ismejo/wrae166)
Supplement: Supplemental_Materials_wrae166 [file supplemental_materials_wrae166.pdf]

Supplemental Materials

Gut microbiota dysbiosis deteriorates immunoregulatory effects of tryptophan via colonic indole and LBP/HTR2B-mediated macrophage function

Lili Jiang[1], Youling Hao[1], Dandan Han[1], Wenjian Dong[1], Aoyu Yang[1], Zhiyuan Sun[1], Yao Ge[1], Shuai Duan[1], Xiuwen Zhang[1], Zhaolai Dai[1,*]

[1]State Key Laboratory of Animal Nutrition and Feeding, College of Animal Science and Technology, China Agricultural University, Beijing 100193, China

*Corresponding author: Zhaolai Dai, State Key Laboratory of Animal Nutrition and Feeding, College of Animal Science and Technology, China Agricultural University, Beijing 100193, China. Email: daizhaolai@cau.edu.cn.

Supplementary Materials and Methods

Reagents

Dextran sodium sulfate (DSS, molecular mass, 36,000–50,000, Cat# 160110) was obtained from MP Biomedicals (Solon, OH, USA). Antibiotics (Abx) including ampicillin sodium salt, neomycin trisulfate salt hydrate, metronidazole, vancomycin hydrochloride, streptomycin, and gentamicin were purchased from Solarbio Life Science (Beijing, China). Dulbecco's modified Eagle's medium, penicillin and streptomycin were purchased from Gibco (Franklin Lakes, NJ, USA). Fetal bovine serum (FBS) was obtained from Pan-Biotech (Aidenbach, Germany). The LBP ELISA kit was purchased from Shanghai Hengyuan Biotechnology (Shanghai, China). Unless otherwise stated, all other chemicals were purchased from Merck Sigma-Aldrich (Shanghai, China).

Antibodies used for Western-blot and flow cytometric analysis

Primary antibodies against TPH1 (Cat# D10C10) and p-NF-κB (Cat# 3036S) were purchased from Cell Signaling Technology (Danvers, MA, USA), and p-NF-κB (Cat# sc-136548) and IκB-α (Cat# sc-1643) were purchased from Santa Cruz Biotechnology (Santa Cruz, CA, USA). Fluorescein-labeled antibodies against CD11b (Cat# 101227), CD11c (Cat# 117308), CD19 (Cat# 115507), CD25 (Cat#

11908), CD3 (Cat# 100203), CD4 (Cat# 100411), CD45 (Cat# 103116), CD8a (Cat# 100733), F4/80 (Cat# 123108), MHCII (Cat# 107613), Ly6G (Cat# 127607), NK1.1 (Cat# 108707), CD11b (Cat# 101227), CD64 (Cat# 139306), CD206 (Cat# 141708), FOXP3 (Cat# 126404), IFN-γ (Cat# 505806), IL-4 (Cat# 504103), and IL-17A (Cat# 506920) were purchased from BioLegend (San Diego, CA, USA).

Analysis of animal growth, disease activity index, spleen weight, and colon length and weight

Body weight, feed intake, and water consumption were recorded daily. Body weight loss, fecal blood content, and stool consistency were recorded for the evaluation of the disease activity index (DAI). The DAI score was evaluated in a double-blinded manner according to previous scoring criteria [1]. Once the mice were euthanized, the spleen was removed and weighed, and the colon was removed for the measurement of colon length.

Cell culture experiment

Mouse RAW264.7 macrophages were maintained at 37°C in a 5% CO_2 humidified incubator in Dulbecco's modified Eagle's medium supplemented with 10% (v/v) heat-inactivated fetal bovine serum and 100 U/mL penicillin and streptomycin. Cells were seeded in six-well plates at a density of 1×10^6 cells/well. After reaching ~60% confluency, the cells were treated with or without 62.5 μmol/L indole for 18 h, followed by 6 h of lipopolysaccharide (LPS) treatment (10 ng/mL) with prior 6-h treatment with 1 μmol/L HTR2B antagonist (SB204741) or PBS. The cells were then harvested for RNA extraction and subsequent analysis. All the cell culture experiments were repeated three times.

RNA extraction and quantitative real-time PCR analysis

Total RNA was extracted from colon tissues using RNAiso Plus (Takara, Beijing, China). The extracted RNA was further purified by the lithium chloride method as described previously [2, 3].The integrity and purity of the RNA were determined using a Nanodrop P330 (Implen, Munich, Germany) and electrophoresis. RNA was then reverse-transcribed using a FastKing RT kit (4992224, TIANGEN BIOTECH). Quantitative real-time PCR was performed using SYBR Premix Ex Taq II (Takara, Beijing, China) with an ABI 7500 real-time PCR detection system (Thermo Fisher, Waltham, MA, USA). The primers used for this study are shown in Supplementary Table S5. GAPDH was utilized as the internal reference. The $2^{-\Delta\Delta Ct}$ method was used for the quantification of gene expression.

Western-blot analysis

Colonic samples were homogenized in liquid nitrogen, and an aliquot of tissue powder was weighed

and used for protein extraction. The protein concentration of the extracted samples was determined by the bicinchoninic acid method [4]. Protein abundance was measured by western blot analysis on the basis of a previous method [5]. The protein bands were detected with ECL Plus detection reagents (Thermo Fisher) and visualized using an Image Quant LAS 4000 mini system (GE Healthcare, Piscataway, NJ, USA). The band intensity of each target protein was compared with that of GAPDH via ImageJ software (NIH, Bethesda, MD, USA).

Colonic morphological examination

For morphological measurements, colonic tissue sections were stained with hematoxylin & eosin (H&E) and evaluated by histological scoring as described previously [3]. The stained sections were visualized by a light microscope equipped with a computer-assisted morphometric system in a double-blinded manner. Three to four sections at a distance of 200 μm were examined per mouse.

Isolation and flow cytometry analysis of immune cells

Immune cells were isolated from the colon and spleen using 40% and 80% Percoll gradient centrifugation method as previously described [6]. Immune cells were collected from the interface after centrifugation at 25°C for 30 min. Peripheral blood lymphocytes were collected from the orbital sinus into tubes with ethylenediaminetetraacetic acid dipotassium salt, and then mixed on a roller for 10 min. Aliquots of the 100 μL cell samples were transferred into 1.5 mL centrifuge tubes for staining and flow cytometry analysis. Before staining, red blood cells were lysed with lysis buffer (Cat# 420301) purchased from BioLegend (San Diego, CA, USA). The above cell suspensions were rinsed and resuspended with 250 μL of cell staining buffer (3% fetal bovine serum in PBS) for subsequent flow cytometry analysis. Analysis was performed using a CytoFLEX flow cytometer (Beckman Coulter, Miami, FL, USA) with the following cell sorting channels: APC-A750, PC5.5, APC, and FITC. The proportions of all immune cells, including $CD11b^+/CD64^+$ macrophages, $CD11b^+/Ly6G^+$ neutrophils, $CD3^+$ T lymphocytes, $CD19^+$ B lymphocytes, $CD3^+/CD4^+$ T helper cells, and $CD3^+/CD8^+$ cytotoxic T cells are expressed as the percentage (%) of the total $CD45^+$ population. $CD25^+/Foxp3^+$ Treg cells are expressed as the percentage of $CD3^+$ populations, and $CD4^+/IFN\text{-}\gamma^+$ Th1 cells, $CD4^+/IL\text{-}4^+$ Th2 cells and $CD4^+/IL\text{-}17A^+$ Th17 cells are expressed as the percentage of $CD4^+$ populations. In this study, M1 macrophages are presented as $F4/80^+$ $CD11c^+$ cells, and M2 macrophages are presented as $F4/80^+$ $CD206^+$ cells.

Analysis of cytokines in serum

The levels of cytokines in the serum of the mice were analyzed with a LEGENDplex™ Mouse Inflammation Panel kit (BioLegend, San Diego, CA, USA) according to the manufacturer's instructions. The incubation mixtures containing cytokines, beads, and antibodies were used for cell sorting via a CytoFLEX flow cytometer (Beckman Coulter, Miami, FL, USA). LEGENDplex^{TM} Data Analysis Software (BioLegend, San Diego, CA, USA) was used to analyze the cytometric bead assay results.

Analyses of Trp metabolites in the serum and colon

For targeted metabolomics analysis of Trp metabolites in the colonic luminal content of mice, approximately 20 mg of sample was weighed in a 1.5 mL tube and mixed with 80 µL of precooled 50% (v/v) methanol: H_2O solution. After vortexing and deproteinization at -20°C for 30 min, the tubes were centrifuged at 15000 × g at 4°C for 10 min. Ten microliter of the supernatant was transferred to a new 1.5 mL centrifuge tube and diluted with 40 µL of 50% methanol solution for further analysis. At the same time, mixed Trp standards (L-kynurenine, kynurenic acid, *N*-acetyltryptophan, tryptamine, indole-3-lactic acid, indole-3-acetic acid, etc.) in acetonitrile were processed with the same method for samples as described above. Then 90 µL of the supernatant was transferred to a new 96-well plate, which was sealed. The processed samples were analyzed by the Metabo-Profile targeted metabolomics technology platform (ACQUITY UPLC-Xevo TQMS, Waters Corp., Milford, MA, USA) for the quantitative analysis of tryptophan metabolites. All chromatographic separations were performed with an ACQUITY UPLC HSS T3 1.8 µM analytical column (2.1 ×100 mm) equipped with an ACQUITY UPLC HSS T3 1.8 µM VanGuard column (2.1×5 mm) (Waters, Milford, MA). The mobile phase consisted of 0.1% (v/v) formic acid H_2O solution and 0.1% (v/v) formic acid acetonitrile solution. Each batch of samples were analyzed in parallel with a reagent blank and quality control (QC) sample. In this analysis, QC was prepared by mixing all the samples and analyzing after every 10 tested samples. The raw data were processed using MassLynx software (version 4.1, Waters, Milford, MA, USA) for compound extraction, peak integration, calibration, and quantification.

In some experiments, the concentrations of Trp metabolites, including 5-HT, indole-3-acetic acid (IAA), indole, 3-methylindole, and tryptamine, in the serum, colon tissue, and colonic luminal content were analyzed by HPLC after precolumn derivatization with *o*-phthaldialdehyde [7]. In brief, 25 µL of serum, 25 mg of colon homogenate or 25 mg of luminal content was deproteinized with 200 µL of extraction solution. The resulting supernatant was further diluted with extraction solution before HPLC analysis [7].

DNA extraction and bacterial 16S rRNA gene sequencing and analysis

Total genomic DNA was extracted from the fecal samples and colonic luminal content samples using an E.Z.N.A soil DNA kit (Omega Bio-Tek, Norcross, GA, USA). The quality of the extracted DNA was detected by 1% agarose gel electrophoresis. The V3-V4 region of the bacterial 16S rRNA gene was amplified by an ABI GeneAmp 9700 PCR thermocycler (ABI, CA, USA) with the primer pairs 338F (5′-ACT CCT ACG GGA GGC AGC AG-3′) and 806R (5′-GGA CTA CHV GGG TWT CTA AT-3′). PCR products were evaluated by 2% agarose gel electrophoresis and recovered using the AxyPrepTM DNA Gel Extraction Kit (Corning, Shanghai, China). The amplified products were detected and quantified by QuantiFluor™, and then sequenced on the Illumina MiSeq PE300 sequencing platform (Illumina, San Diego, USA) according to the methods of Majorbio Bio-Pharm Technology Co. Ltd. (Shanghai, China). The original data were analyzed by QIIME2 on the Majorbio cloud platform. The raw reads were first quality controlled by fastp (version 0.19.6), filtered and spliced by FLASH (version 1.2.7) to optimize the data, which were processed through the sequential noise reduction method (DADA2/Deblur, etc.) to obtain the ASV (amplicon sequence variant) representative sequence and abundance information. The silva138/16S-bacteria database was used to sort ASVs with 100% sequence similarity as the threshold. α- and β-diversity were also determined by QIIME 2. Principal coordinate analysis (PCoA) based on weighted Bray−Curtis distances was carried out using R software (version 3.3.1). The differential microbiota between all groups were identified using linear discriminant analysis effect size (LEfSe) analysis (http://huttenhower.sph.harvard.edu/galaxy/root?tool_id=lefse_upload) [8]. Only bacterial taxa with a linear discriminant analysis (LDA) threshold of 2.0 and an average relative abundance greater than 0.01% were included in the results. Correlations between differential bacterial taxa and levels of inflammatory cytokines, HTRs, and Trp metabolites were analyzed by Spearman's correlation analysis using the pheatmap package of R software (version 3.3.1). Rarefaction curves and rank-abundance curves showed that the sequencing depth was sufficient for the analysis of the fecal microbiota composition (Supplementary Fig. S5A, S5B).

Metagenomic sequencing, library construction and functional analysis

Total genomic DNA was extracted from the colonic luminal content samples using an E.Z.N.A soil DNA kit (Omega Bio-Tek, Norcross, GA, USA).The quality of the extracted DNA was analyzed by 1% agarose gel electrophoresis. The DNA was then fragmented to approximately 400 bp in length by an M220 Focused-ultrasonicator (Covaris, Woburn, MA, USA). Adapter ligation, cleanup, and

enrichment were carried out with the NEXTFLEX™ Rapid DNA-Seq Kit (PerkinElmer, USA). Shotgun metagenomic sequencing was performed on an Illumina NovaSeq/HiSeq Xten platform (Majorbio Bio-Pharm Technology Co., Ltd., Shanghai, China). Before analysis, Fastp software (version 0.20.0) was used to conduct quality control on the adapter sequences, low-quality bases, N bases (uncertain bases) and short sequences to obtain high-quality paired-end reads and single-end reads. Contaminated reads with high similarity to the *Mus musculus* genome were removed by the Burrows−Wheeler Aligner (version 0.7.17). The short sequence obtained after quality control was assembled by Megahit (version 1.1.2).

A nonredundant gene catalog was constructed using CD-HIT (version 4.7) with 90% sequence identity and 90% coverage identity [9]. High-quality reads were mapped back to the established nonredundant gene catalog with 95% identity using SOAPaligner (soap2.21 release) to estimate gene abundance within each sample. The original sequence was optimized by splitting, mass shearing and pollution removal. The optimized sequences were then used for splicing assembly and gene prediction with Prodigal (version 2.6.3). The obtained genes were annotated and classified in terms of species and function using publicly available databases including the Kyoto Encyclopedia of Genes and Genomes (KEGG) and carbohydrate-active enzymes (CAZyme).

Transcriptome analysis and validation of gene expression

Total RNA from colon tissue was extracted using RNAiso Plus (Takara, Beijing, China) and reverse-transcribed using the PrimeScript™ RT reagent Kit with gDNA Eraser (Takara, Beijing, China). RNA-seq was performed following Illumina® Stranded mRNA Prep, Ligation from Illumina (San Diego, CA) to construct RNA libraries, and then sequencing was performed using Illumina NovaSeq Xplus (San Diego, CA) to obtain high-quality clean data, which were subsequently aligned to the reference genome in orientation mode using HISAT version 2.1.0. The mapped reads of each sample were assembled by StringTie (version 2.1.2). The *Mus musculus* reference genome (GRCm39) (http://asia.ensembl.org/Mus-musculus/Info/Index) and gene model annotation files were downloaded from the NCBI database. DEGs were identified through pairwise comparisons between every two stages by DESeq2 (version 1.24.0). DEGs with a $|\log_2 FC| > 1.5$ and a Q value ≤ 0.05 were regarded as significantly different. Functional enrichment analysis was performed based on a Bonferroni-corrected P value ≤ 0.05 by GOATOOLS (version 0.6.5) and KOBAS (version 2.1.1) using the KEGG database.

Supplementary References

1. Lu F, Fernandes SM, Davis AE, 3rd. The role of the complement and contact systems in the dextran sulfate sodium-induced colitis model: the effect of C1 inhibitor in inflammatory bowel disease. *Am J Physiol Gastrointest Liver Physiol*. 2010;**298**:G878-83.
2. Viennois E, Chen F, Laroui H *et al.* Dextran sodium sulfate inhibits the activities of both polymerase and reverse transcriptase: lithium chloride purification, a rapid and efficient technique to purify RNA. *BMC Res Notes*. 2013;**6**:360.
3. Hao Y, Jiang L, Han D *et al. Limosilactobacillus mucosae* and *Lactobacillus amylovorus* protect against experimental colitis via upregulation of colonic 5-hydroxytryptamine receptor 4 and transforming growth factor-β2. *J Nutr*. 2023;**153**:2512-22.
4. Lei J, Feng D, Zhang Y *et al.* Regulation of leucine catabolism by metabolic fuels in mammary epithelial cells. *Amino Acids*. 2012;**43**:2179-89.
5. Wang H, Zhang C, Wu G *et al.* Glutamine enhances tight junction protein expression and modulates corticotropin-releasing factor signaling in the jejunum of weanling piglets. *J Nutr*. 2015;**145**:25-31.
6. Qiu Z, Sheridan BS. Isolating lymphocytes from the mouse small intestinal immune system. *J Vis Exp*. 2018:57281.
7. Dai Z, Sun S, Chen H *et al.* Analysis of tryptophan and its metabolites by high-performance liquid chromatography. *Methods Mol Biol*. 2019;**2030**:131-42.
8. Segata N, Izard J, Waldron L *et al.* Metagenomic biomarker discovery and explanation. *Genome Biol*. 2011;**12**:R60.
9. Li R, Yu C, Li Y *et al.* SOAP2: an improved ultrafast tool for short read alignment. *Bioinformatics*. 2009;**25**:1966-7.

Supplementary Table S1. Metabolomics analysis of the effects of Trp treatment and timing of antibiotic exposure on Trp metabolites in the colonic luminal content of mice with DSS-induced colitis[†]

Trp metabolites (nmol/g)	Ctrl	DSS	Trp+DSS	Trp+Abx+DSS	Trp+DSS+Abx	*P* value
L-Kynurenine	1.14 ± 0.32^{a}	0.32 ± 0.05^{b}	0.33 ± 0.09^{b}	0.21 ± 0.04^{b}	0.26 ± 0.07^{b}	< 0.01
Kynurenic acid	15.4 ± 0.42^{a}	1.52 ± 0.10^{c}	2.72 ± 0.35^{b}	1.04 ± 0.30^{c}	1.40 ± 0.29^{c}	< 0.001
Quinolinic acid	1.48 ± 0.14^{a}	0.70 ± 0.08^{c}	0.93 ± 0.08^{bc}	0.99 ± 0.16^{bc}	1.28 ± 0.19^{ab}	< 0.01
Nicotinamide	2.99 ± 0.36^{c}	18.8 ± 3.93^{ab}	10.3 ± 2.20^{bc}	14.9 ± 3.60^{ab}	23.7 ± 4.86^{a}	< 0.01
Xanthurenic acid	7.15 ± 0.69^{a}	0.81 ± 0.18^{b}	2.00 ± 0.41^{b}	1.62 ± 0.12^{b}	1.94 ± 0.38^{b}	< 0.001
Nicotinic acid	344 ± 43.9^{a}	31.1 ± 6.21^{b}	34.5 ± 6.98^{b}	28.9 ± 4.83^{b}	54.9 ± 8.39^{b}	< 0.001
Picolinic acid	9.73 ± 2.06^{a}	1.23 ± 0.17^{c}	0.67 ± 0.12^{c}	2.03 ± 0.27^{bc}	4.51 ± 0.69^{b}	< 0.001
N-Acetyltryptophan	1.63 ± 0.37^{a}	0.23 ± 0.04^{b}	0.52 ± 0.14^{b}	0.34 ± 0.08^{b}	0.98 ± 0.25^{ab}	< 0.001
Tryptamine	0.38 ± 0.06^{a}	0.07 ± 0.01^{b}	0.08 ± 0.02^{b}	0.18 ± 0.12^{ab}	0.03 ± 0.01^{b}	< 0.01
Indole-3-lactic acid	4.74 ± 0.56^{b}	0.77 ± 0.19^{c}	3.50 ± 0.65^{b}	12.0 ± 2.10^{a}	12.5 ± 1.68^{a}	< 0.001
Indole-3-acetic acid	22.4 ± 3.79^{a}	2.16 ± 0.49^{c}	5.10 ± 0.99^{b}	2.73 ± 0.63^{c}	1.86 ± 0.26^{c}	< 0.001
Indole-3-propionic acid	4.71 ± 0.42^{a}	0.18 ± 0.06^{c}	0.99 ± 0.05^{b}	0.08 ± 0.01^{c}	0.08 ± 0.02^{c}	< 0.001
Indole-3-carboxaldehyde	4.23 ± 0.72^{a}	0.16 ± 0.02^{b}	0.33 ± 0.15^{b}	0.21 ± 0.02^{b}	0.28 ± 0.08^{b}	< 0.001
Indole-3-carboxylic acid	1.07 ± 0.15^{a}	0.06 ± 0.01^{b}	0.12 ± 0.01^{b}	0.08 ± 0.02^{b}	0.10 ± 0.01^{b}	< 0.001

[†]Values are means ± SEMs. a-c, values within a row without a common superscript letter differ, $P < 0.01$.

Ctrl, control; Abx, antibiotic; DSS, dextran sodium sulfate; Trp, tryptophan.

Supplementary Table S2. Effects of Trp treatment and timing of antibiotic exposure on the α-diversity of the fecal microbiota of mice with DSS-induced colitis[†]

α-Diversity	DSS	Trp+DSS	Trp+Abx+DSS	Trp+DSS+Abx	*P* value
Sobs	259 ± 31.1[a]	193 ± 11.5[a]	90.8 ± 15.6[b]	63.6 ± 7.98[b]	< 0.01
Ace	267 ± 32.9[a]	197 ± 12.6[b]	92.5 ± 15.8[c]	65.4 ± 8.14[c]	< 0.01
Chao	267 ± 33.3[a]	196 ± 12.3[b]	91.9 ± 15.8[c]	64.4 ± 8.14[c]	< 0.01
Shannon	4.05 ± 0.14[a]	3.59 ± 0.07[ab]	3.00 ± 0.20[bc]	2.78 ± 0.16[c]	< 0.01

[†]Values are means ± SEMs. a-c, values within a row without a common superscript letter differ, $P < 0.01$.
Abx, antibiotic; DSS, dextran sodium sulfate; Trp, tryptophan.

Supplementary Table S3. Comparison of the α-diversity of fecal microbiota of mice before and after antibiotic treatment[†]

α-Diversity	Abx-Day 0	Abx-Day 14	*P* value
Sobs	170 ± 16.8	242 ± 26.9	< 0.05
Ace	187 ± 19.2	279 ± 31.0	< 0.05
Chao	187 ± 18.7	273 ± 30.2	< 0.05
Shannon	3.93 ± 0.17	3.99 ± 0.20	0.82

[†]Values are means ± SEMs.

Abx, antibiotic.

Supplementary Table S4. Comparison of the α-diversity of fecal microbiota of mice after FMT with donor microbiota from mice of different groups and DSS treatment†

α-Diversity	FMT-Ctrl	FMT-Trp	FMT-SFF-Trp	FMT-Trp+Abx	*P* value
Sobs	232 ± 30.2^{a}	136 ± 33.2^{b}	99.2 ± 17.4^{b}	77.7 ± 4.33^{b}	< 0.01
Ace	233 ± 30.8^{a}	138 ± 33.5^{b}	100 ± 17.4^{b}	77.7 ± 4.37^{b}	< 0.01
Chao	233 ± 30.9^{a}	137 ± 33.4^{b}	99.5 ± 17.4^{b}	77.7 ± 4.33^{b}	< 0.01
Shannon	3.93 ± 0.58^{a}	3.35 ± 0.69^{ab}	2.96 ± 0.39^{b}	2.90 ± 0.24^{b}	< 0.01

†Values are means ± SEMs. a-b, values within a row without a common superscript letter differ, $P < 0.01$.

Abx, antibiotic; Ctrl, control; DSS, dextran sodium sulfate; FMT, fecal microbiota transplantation; FMT-Ctrl, mice transplanted with the fecal microbiota of control mice; FMT-Trp, mice transplanted with fecal microbiota from Trp-treated mice; FMT-SFF-Trp, mice transplanted with sterile fecal filtrate (SFF) from Trp-treated mice; FMT-Trp+Abx group, mice transplanted with fecal microbiota from Trp+Abx-treated mice.

Supplementary Table S5. Primers used in this study

Genes	Primer sequence (5'→3')	Product size, bp
TNF-α	F: CCACCACGCTCTTCTGTCTA	101
	R: GGTCTGGGCCATAGAACTGA	
IL-1α	F: GCCCGTGTTGCTGAAGGA	74
	R: AGAAGAAAATGAGGTCGGTCTCA	
IL-1β	F: CAGGCAGGCAGTATCACTCA	89
	R: TGTCCTCATCCTGGAAGGTC	
IL-6	F: CTGATGCTGGTGACAACCAC	143
	R: TCCACGATTTCCCAGAGAAC	
IL-10	F: CCAGGGAGATCCTTTGATGA	96
	R: AACTGGCCACAGTTTTCAGG	
IL-17α	F: ACGTTTCTCAGCAAACTTAC	127
	R: CCCCTTTACACCTTCTTTTC	
IL-22	F: CCGAGGAGTCAGTGCTAAGG	106
	R: CATGTAGGGCTGGAACCTGT	
IFN-γ	F: GCTCTGAGACAATGAACGCTACAC	149
	R: TTCTTCCACATCTATGCCACTTGAG	
CCL2	F: CCCAATGAGTAGGCTGGAGA	125
	R: TCTGGACCCATTCCTTCTTG	
CCL3	F: ACCTGGAACTGAATGCCTGA	161
	R: GTCCCTCGATGTGGCTACTT	
CCL8	F: GTCACCTGCTGCTTTCATGT	119
	R: CCCTGCTTGGTCTGGAAAAC	
CCL20	F: ACTGTTGCCTCTCGTACATACA	176
	R: GAGGAGGTTCACAGCCCTTTT	
iNOS	F: ACATCGACCCGTCCACAGTAT	177
	R: CAGAGGGGTAGGCTTGTCTC	
Arg-1	F: GAATCTGCATGGGCAACC	73
	R: GAATCCTGGTACATCTGGGAAC	
CD206	F: TCTTTGCCTTTCCCAGTCTCC	241
	R: TGACACCCAGCGGAATTTC	
TPH1	F: ACGTTCCTCTCTTGGCTGAA	106
	R: TAGCACGTTGCCAGTTTTTG	
SERT	F: TCACATATGCGGAGGCAATA	91
	R: CTATCCAAACCCAGCGTGAT	
HTR1A	F: CCGTGAGAGGAAGACAGTGAAGAC	176
	R: GGTTGAGCAGGGAGTTGGAGTAG	
HTR2A	F: GAACCAACCTCTCCTGCGAA	146
	R: GGACACTGCCATGATGACCA	
HTR2B	F: GAGGCTCCGAAGTTCAACCA	118
	R: CGGCCCGTTCTGCTATATGT	
HTR4	F: TAATGTTGGGAGGCTGCTGG	193
	R: GGGATGTAGAAGGCCACCAC	
HTR7	F: AAGTTCTCAGGCTTCCCACG	92
	R: TTCGCACACTCTTCCACCTC	
LBP	F: GCAGCCGCATTTGTGATTTG	85
	R: TTCGACTCGATCTGGTTGTGG	
IκB-α	F: TGAAGGACGAGGAGTACGAGC	146
	R: TTCGTGGATGATTGCCAAGTG	
NF-κB	F: AGCGCGGGGACTATGACTT	121
	R: GCCCGGTTATCAAAAATCGGAT	
GAPDH	F: ATGGGAAGCTTGTCATCAACG	115
	R: AAGACACCAGTAGACTCCACG	

Supplementary Figures

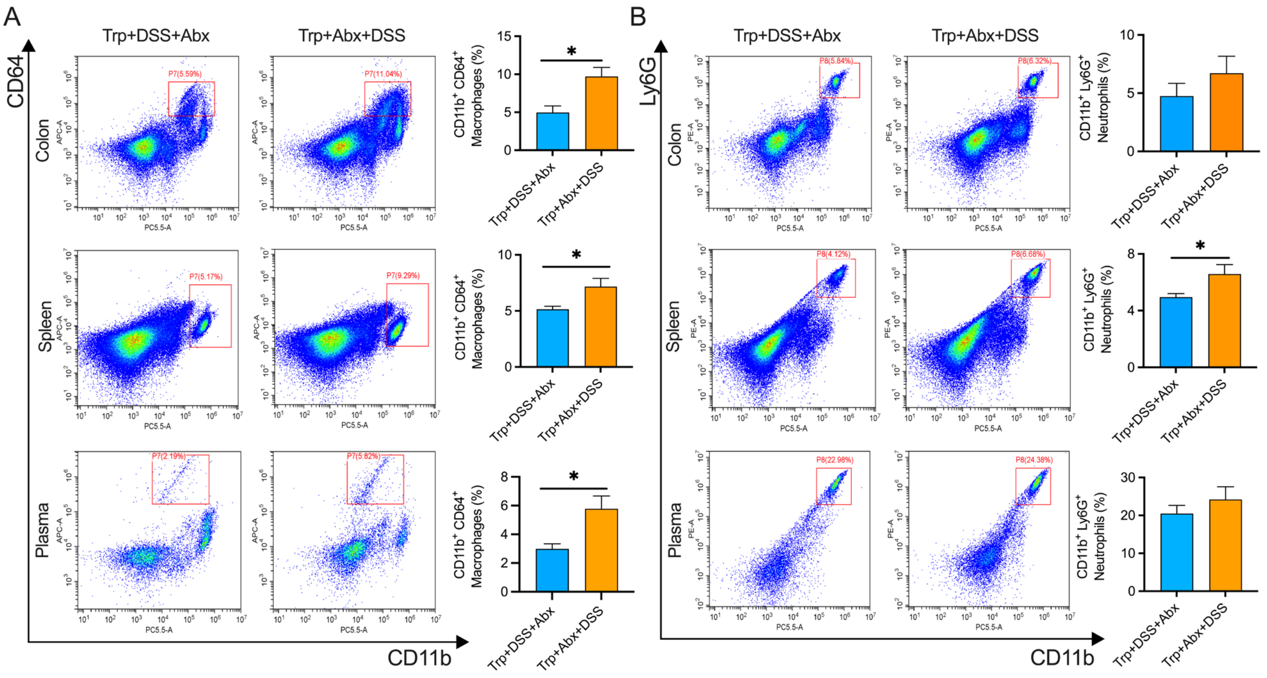

Supplementary Fig. S1 Effects of Trp treatment and antibiotic exposure on the proportions of macrophages and neutrophils in the colon, spleen and plasma of mice with DSS-induced colitis. The proportions of $CD11b^{+}CD64^{+}$ macrophages (**A**) and $CD11b^{+}Ly6G^{+}$ neutrophils (**B**) are shown. The data are the means ± SEMs. *$P < 0.05$.

Abx, antibiotic; DSS, dextran sodium sulfate; Trp, tryptophan.

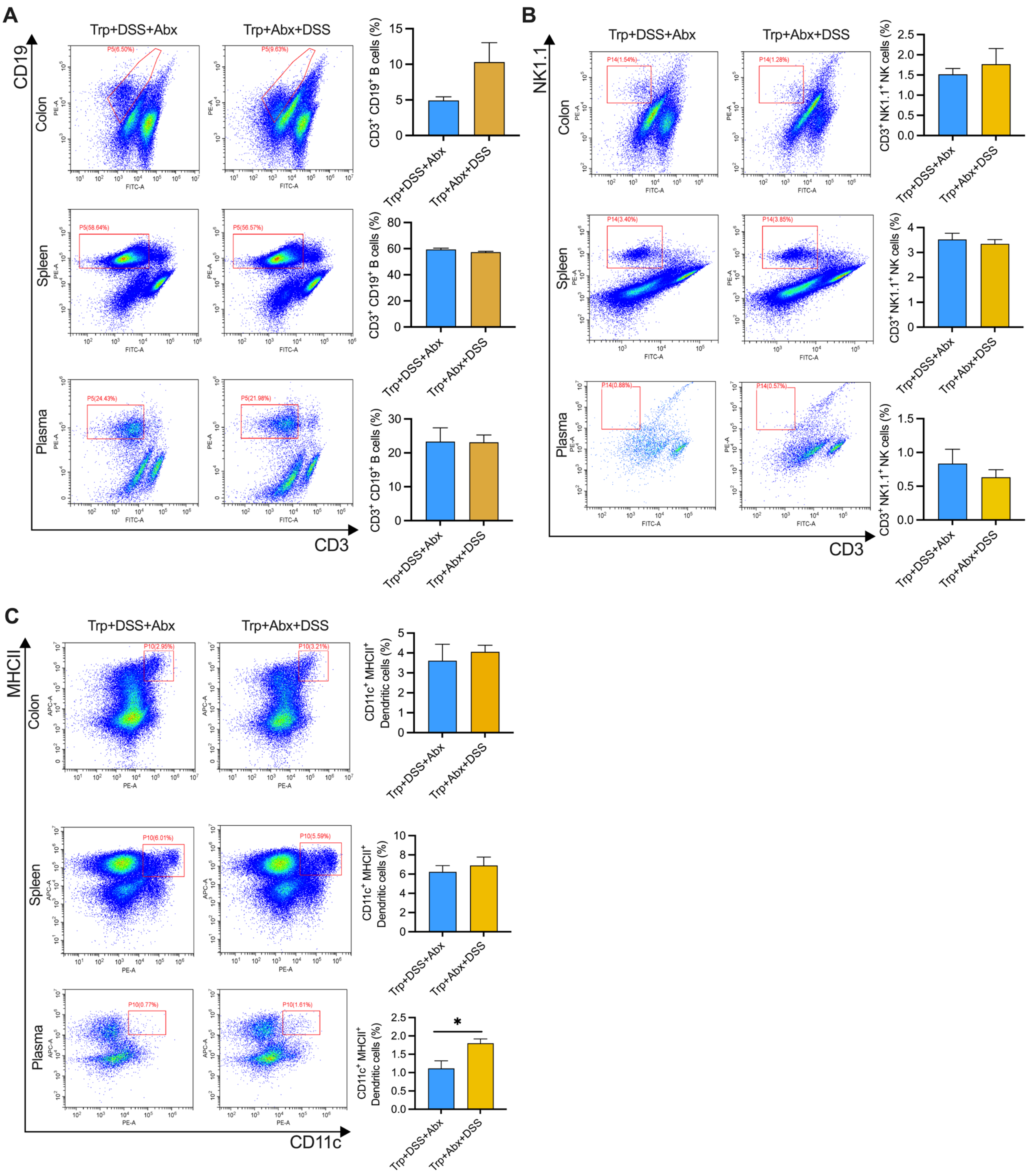

Supplementary Fig. S2 Effects of Trp treatment and antibiotic exposure on the proportions of B cells, NK cells and dendritic cells in the colon, spleen and plasma of mice with DSS-induced colitis. The proportions of $CD3^+CD19^+$ B cells (**A**), $CD3^+NK1.1^+$ NK cells (**B**), and $CD11c^+MHCII^+$ dendritic cells (**C**) are shown. Data are means ± SEMs. * $P < 0.05$.

Abx, antibiotic; DSS, dextran sodium sulfate; NK cells, natural killer cells; Trp, tryptophan.

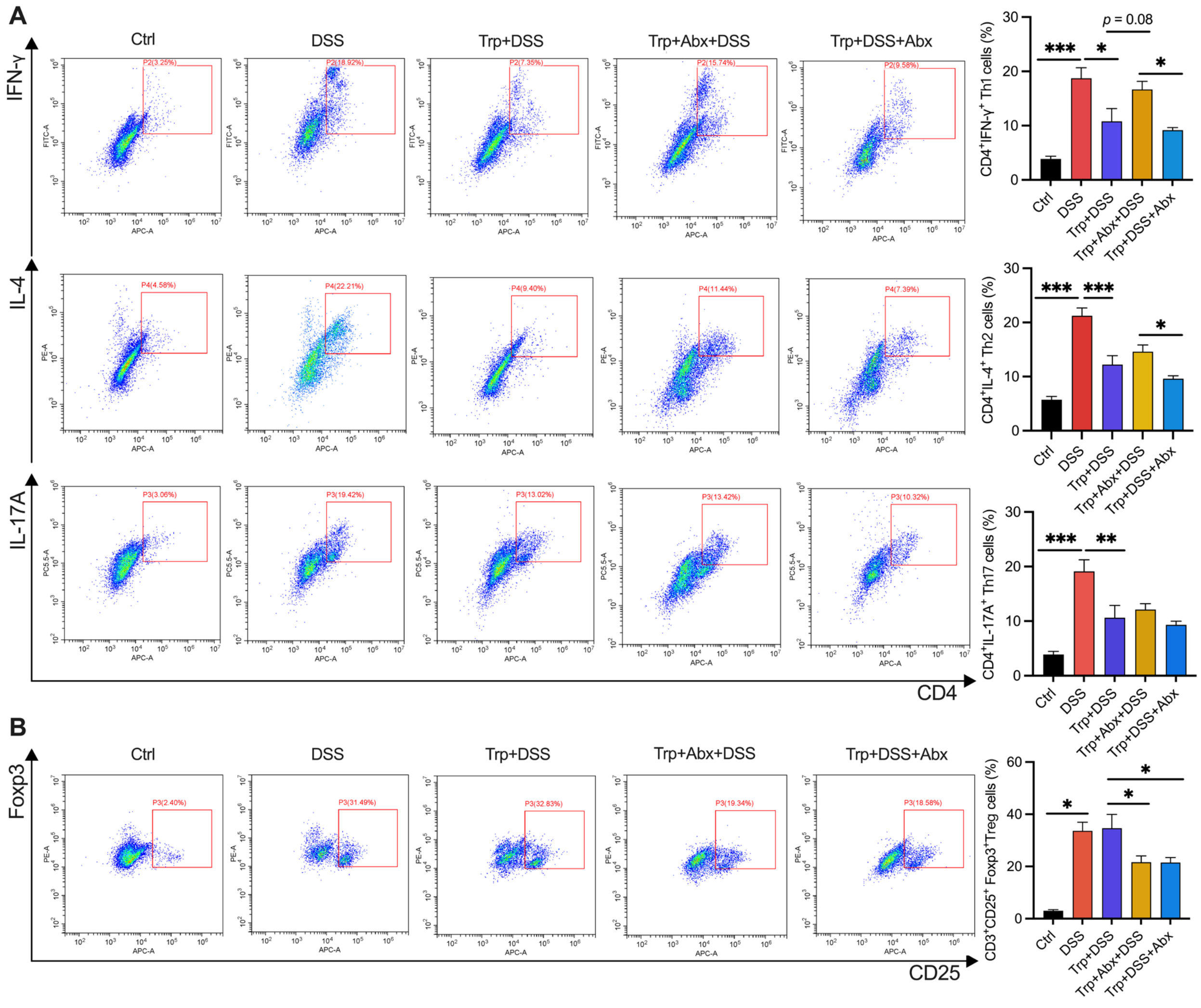

Supplementary Fig. S3 Effects of Trp treatment and antibiotic exposure on the proportions of T helper cell and regulatory T cell subtypes in the colon of mice with DSS-induced colitis. The proportions of $CD4^{+}IFN\text{-}\gamma^{+}$ Th1 cells, $CD4^{+}IL\text{-}4^{+}$ Th2 cells, and $CD4^{+}IL\text{-}17A^{+}$ Th17 cells (**A**), and $CD4^{+}CD25^{+}Foxp3^{+}$ Treg cells (**B**) are shown. The data are the means ± SEMs. * $P < 0.05$, ** $P < 0.01$, *** $P < 0.001$.

Abx, antibiotic; Ctrl, control; DSS, dextran sodium sulfate; Th cells, T helper cells; Treg cells, regulatory T cells; Trp, tryptophan.

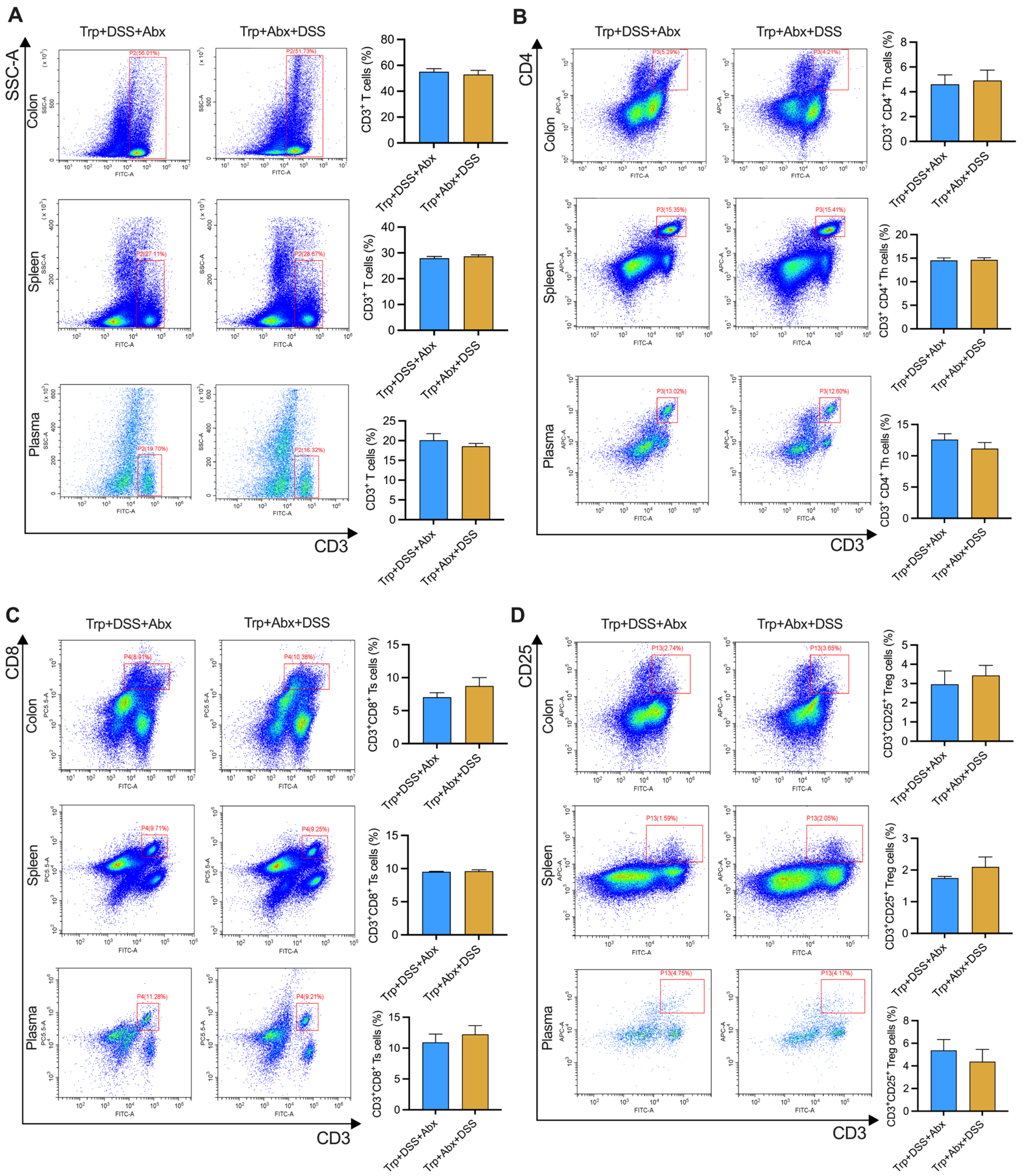

Supplementary Fig. S4 Effects of Trp treatment and antibiotic exposure on the proportions of CD3⁺ T lymphocyte subtypes in the colon, spleen and plasma of mice with DSS-induced colitis. The proportions of CD3⁺ T cells (**A**), CD3⁺CD4⁺ Th cells (**B**), CD3⁺CD8⁺ Ts cells (**C**), and CD3⁺CD25⁺ Treg cells (**D**) are shown. The data are the means ± SEMs.

Abx, antibiotic; DSS, dextran sodium sulfate; Th cells, T helper cells; Treg cells, regulatory T cells; Trp, tryptophan; Ts cells, T suppressor cells.

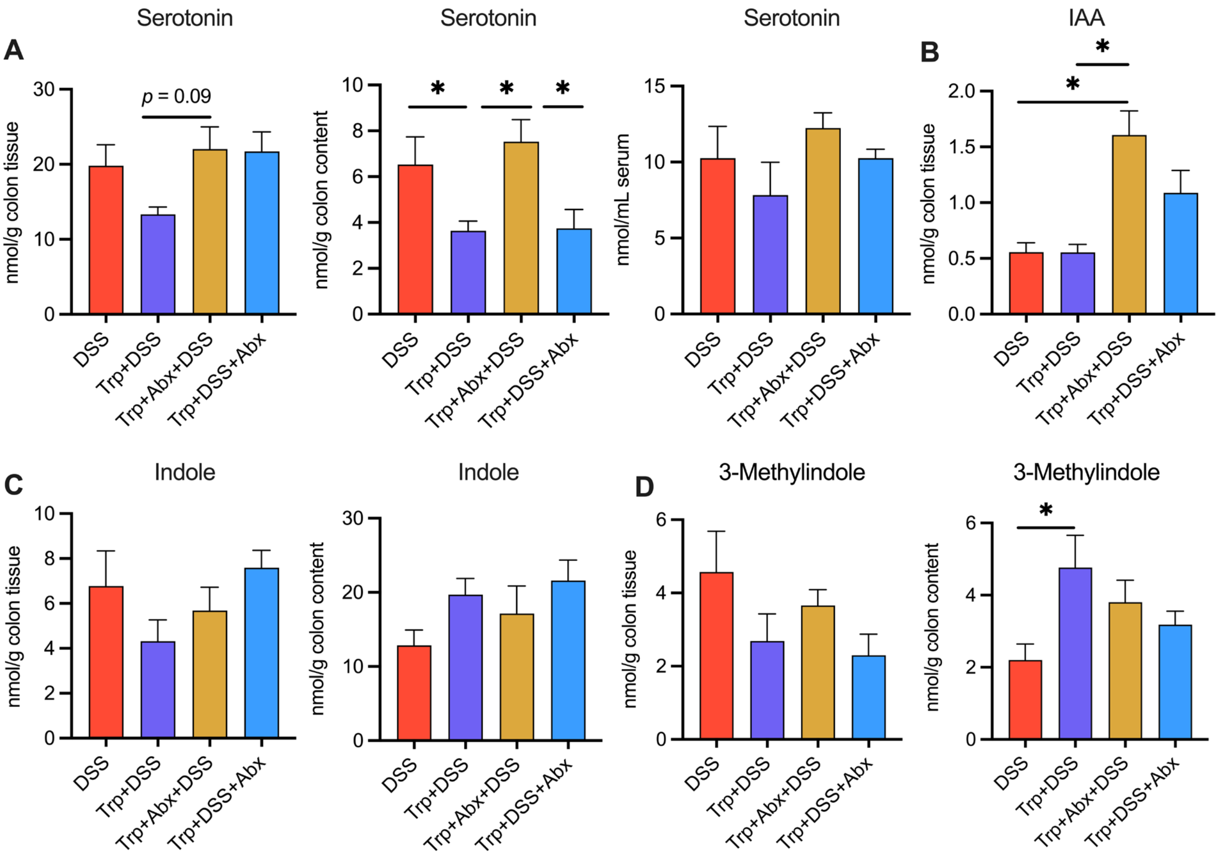

Supplementary Fig. S5 Effects of Trp treatment and antibiotic exposure on Trp metabolites in the colonic tissue, colonic luminal content and serum of DSS-treated mice. The levels of serotonin (**A**), IAA (**B**), indole (**C**), and 3-methylindole (**D**) in tissues and luminal contents are shown. The data are the means ± SEMs. * $P < 0.05$.

Abx, antibiotic; DSS, dextran sodium sulfate; IAA, indole-3-acetic acid; Trp, tryptophan.

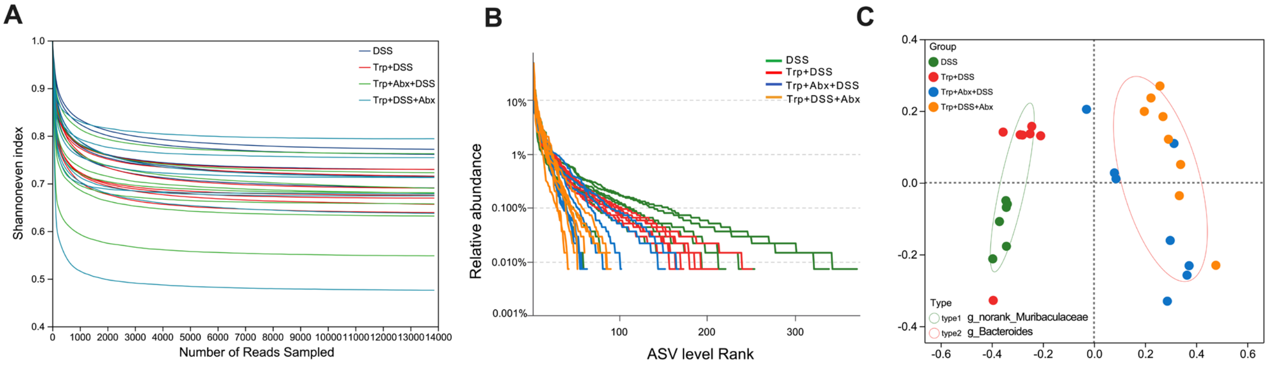

Supplementary Fig. S6 Effects of Trp treatment and antibiotic exposure on the gut microbiota diversity of mice with DSS-induced colitis. A Rarefaction curves analyzed by the Shannon even index. **B** Rank-abundance curves at the ASV level. **C** Enterotype analysis at the genus level.

Abx, antibiotic; ASV, amplicon sequence variant; DSS, dextran sodium sulfate; Trp, tryptophan.

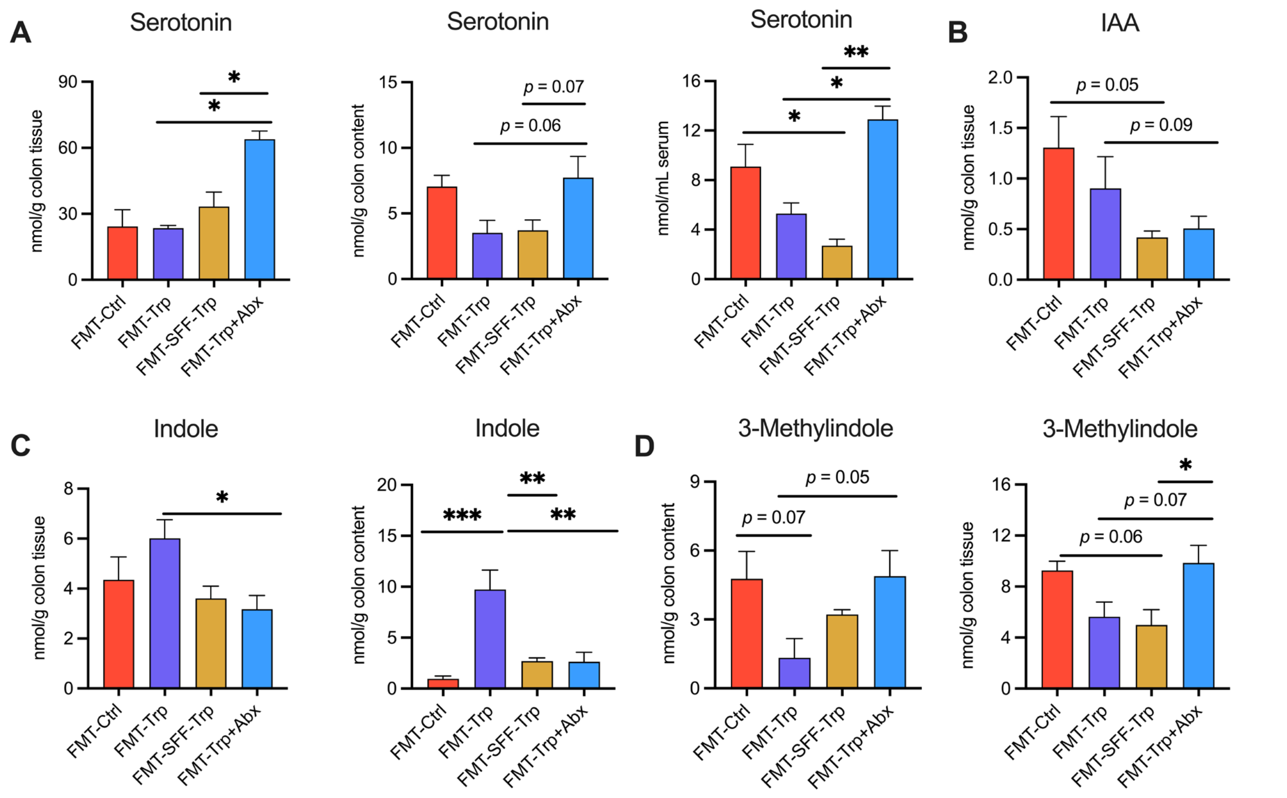

Supplementary Fig. S7 Effects of FMT with donor microbiota from mice in different groups on Trp metabolites in the colon of DSS-treated mice. The levels of serotonin (**A**), IAA (**B**), indole (**C**), and 3-methylindole (**D**) in tissues and luminal contents are shown. The data are the means ± SEMs. * $P < 0.05$, ** $P < 0.01$, *** $P < 0.001$.

Abx, antibiotic; Ctrl, control; DSS, dextran sodium sulfate; FMT, fecal microbiota transplantation; IAA, indole-3-acetic acid; Trp, tryptophan.

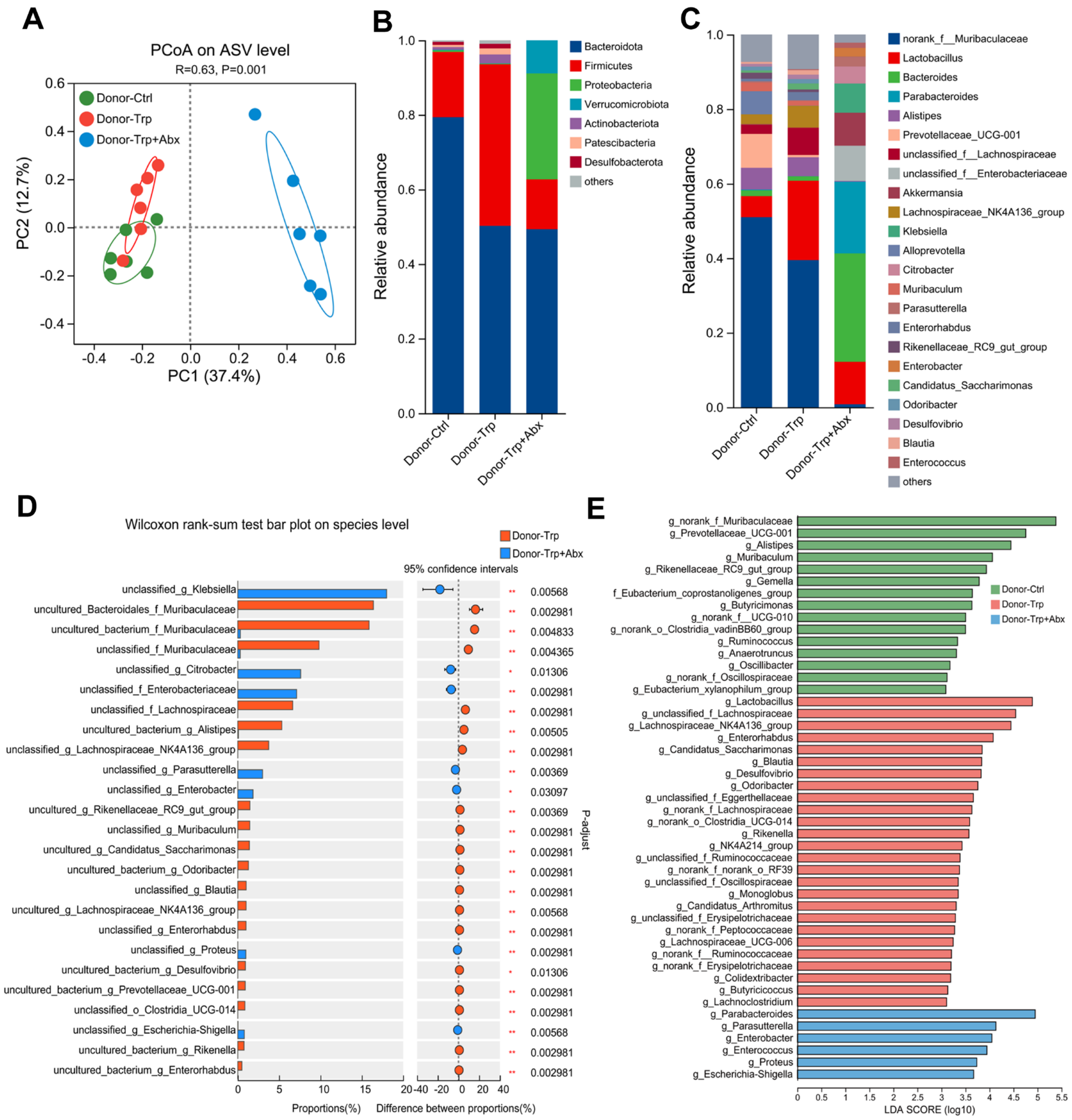

Supplementary Fig. S8 Comparison of the differences in the fecal microbiota composition of the donor mice in the FMT experiment. A Principal coordinate analysis plots of fecal microbiota among treatment groups using the Bray–Curtis distance method. Relative abundance of the fecal microbiota composition at the phylum level (**B**) and genus level (**C**). **D** Pairwise group comparisons of the differential gut microbiota between the Donor-Trp and Donor-Trp+Abx groups based on the Wilcoxon rank-sum test. **E** LEfSe analysis of differential fecal microbiota at the genus level (LDA > 3). * $P < 0.05$, ** $P < 0.01$.

Abx, antibiotic; ASV, amplicon sequence variant; Ctrl, control; LDA, linear discriminant analysis; Trp, tryptophan.

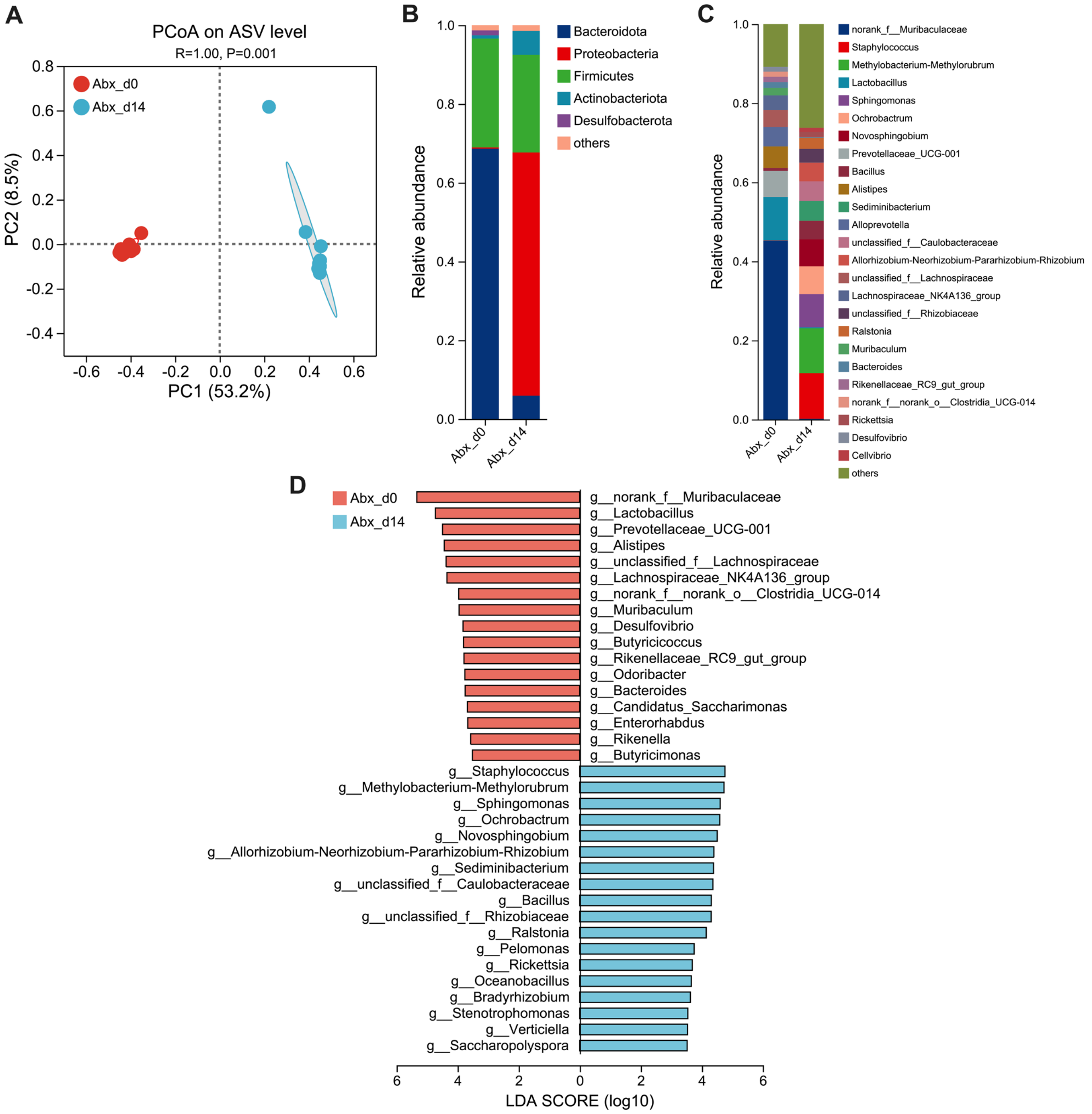

Supplementary Fig. S9 Changes in composition of the fecal microbiota after 14 days of antibiotic treatment. **A** Principal coordinate analysis plots of the fecal microbiota in mice before and after antibiotic treatment based on the Bray–Curtis distance. The fecal microbiota compositions at the phylum level (**B**) and genus level (**C**). **D** LEfSe analysis of differential enrichment of the fecal microbiota at genus level (LDA > 3).

Abx, antibiotic; ASV, amplicon sequence variant; LDA, linear discriminant analysis.

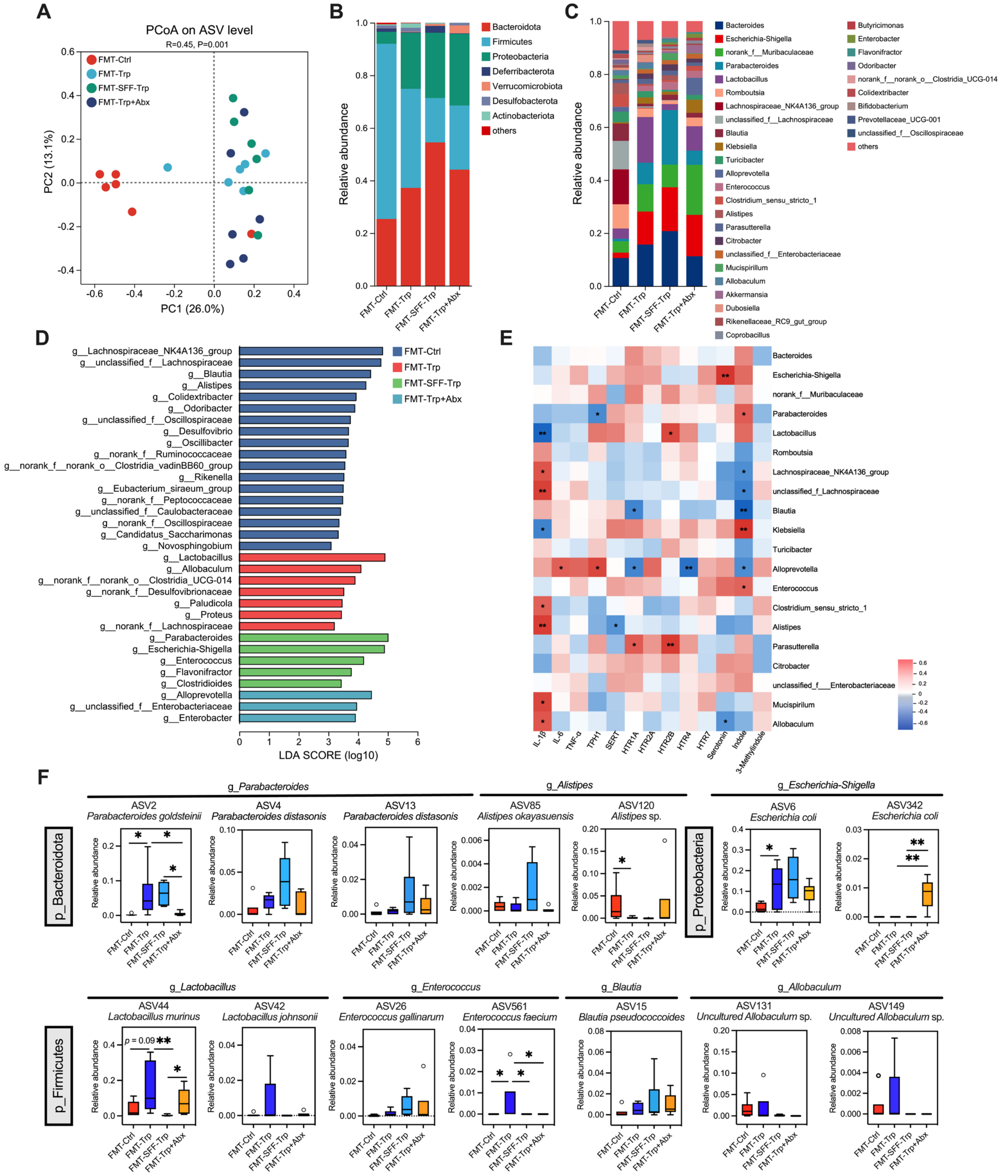

Supplementary Fig. S10 Effects of FMT with donor microbiota from mice in different groups on the gut microbiota composition of recipient mice with DSS-induced colitis. A Principal coordinate analysis plots of the colonic microbiota in mice in different treatment groups based on the Bray–Curtis distance. The relative abundance of colonic bacteria at the phylum level (**B**) and genus level (**C**). **D** LEfSe analysis of the differential enrichment of the colonic microbiota at the genus level (LDA > 3). **E** Spearman correlation analysis between the colonic microbiota and anti-inflammatory cytokines, 5-HT metabolism, HTRs, and indole metabolites. **F** Box plot comparison of the top 20 differential ASVs among the treatment groups. **P* < 0.05, ***P* < 0.01.

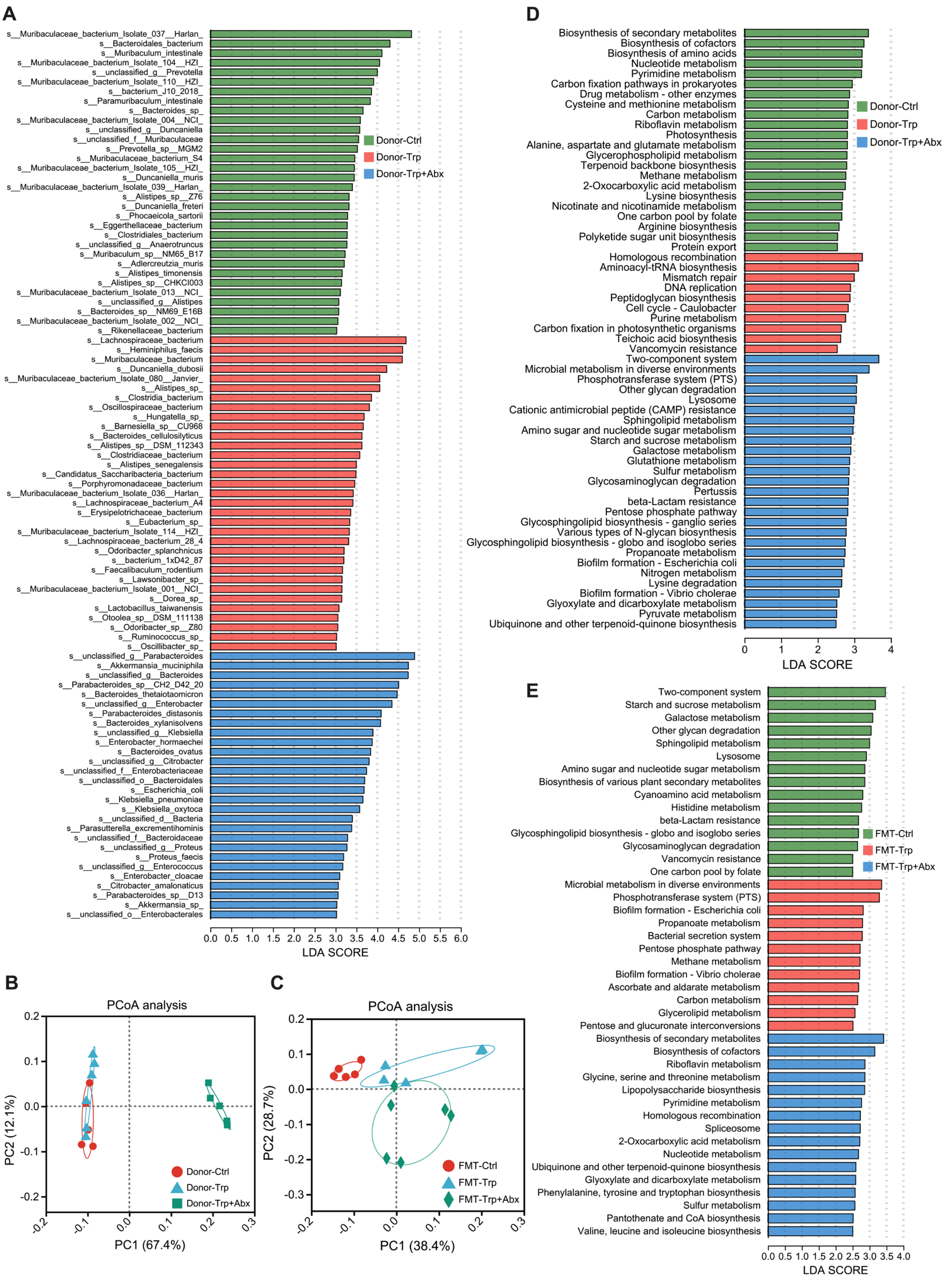

Supplementary Fig. S11 Metagenomic analysis of the composition and function of the fecal microbiota of donor mice and the colonic microbiota of recipient mice after FMT and DSS treatment. A LEfSe analysis of differential enrichment of fecal microbiota at the species level in donor mice (LDA > 3). Principal coordinate analysis based on the Bray–Curtis distances of the

KEGG KO data of the donor mice (**B**) or recipient mice after FMT (**C**). LEfSe analysis (LDA > 2.5) of the differentially enriched KEGG pathways of the microbiome from the donor mice (**D**) or the recipient mice after FMT (**E**).
Abx, antibiotic; Ctrl, control; FMT, fecal microbiota transplantation; KEGG, Kyoto Encyclopedia of Genes and Genomes; LDA, linear discriminant analysis; Trp, tryptophan.

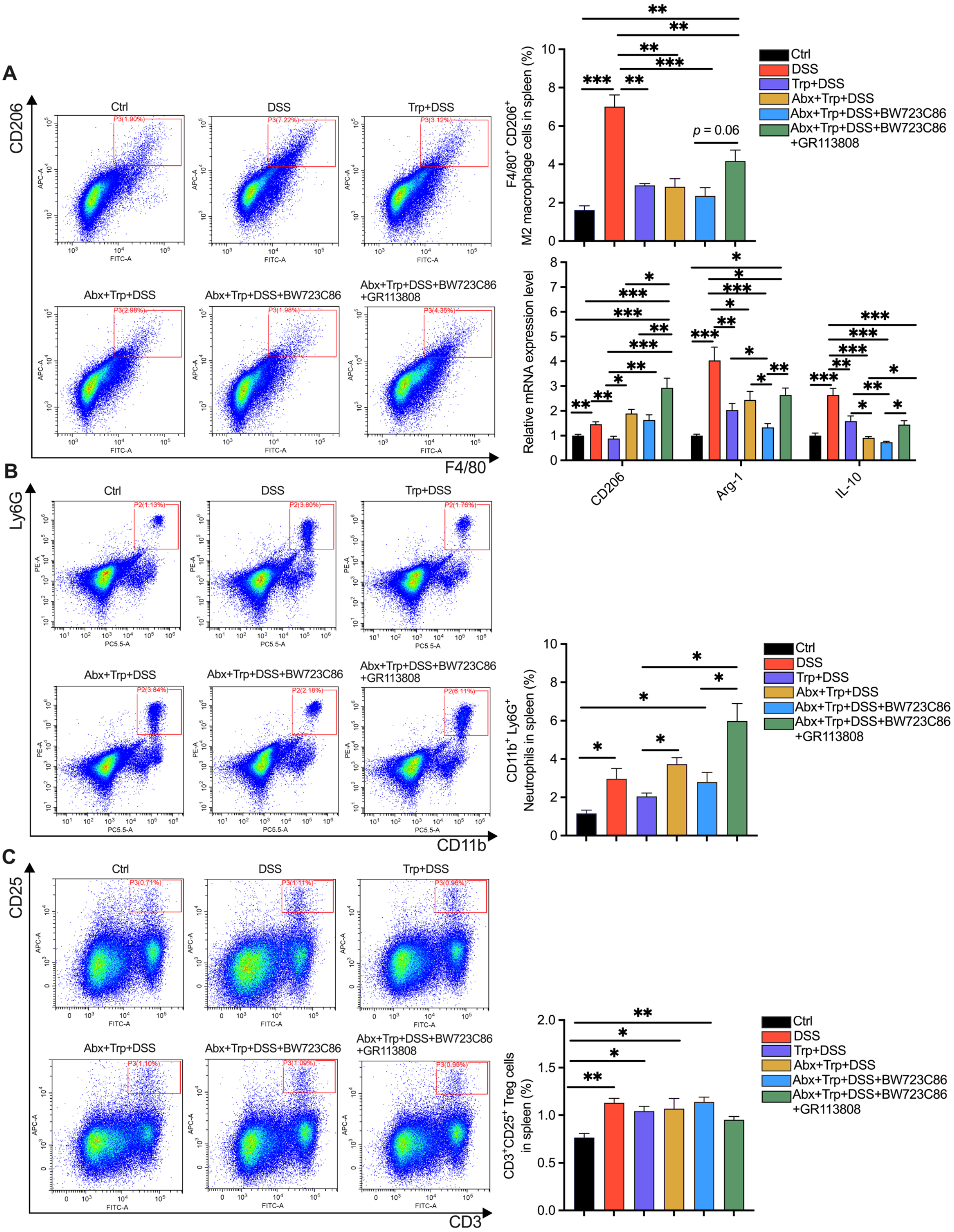

Supplementary Fig. S12 Effects of HTR2B activation on the abundances of M2 macrophages, neutrophils and Treg cells in the spleen of antibiotic- and Trp-treated mice with DSS-induced colitis. The proportion of F4/80^{+} CD206^{+} M2 macrophages and expressions of related marker genes (**A**), proportion of CD11b^{+}Ly6G^{+} neutrophils (**B**), and proportion of CD3^{+}CD25^{+} Treg cells (**C**) are shown. Data are means ± SEMs. * $P < 0.05$, ** $P < 0.01$, *** $P < 0.001$.

Abx, antibiotic; Ctrl, control; DSS, dextran sodium sulfate; Treg lymphocytes, T regulatory lymphocytes; Trp, tryptophan.

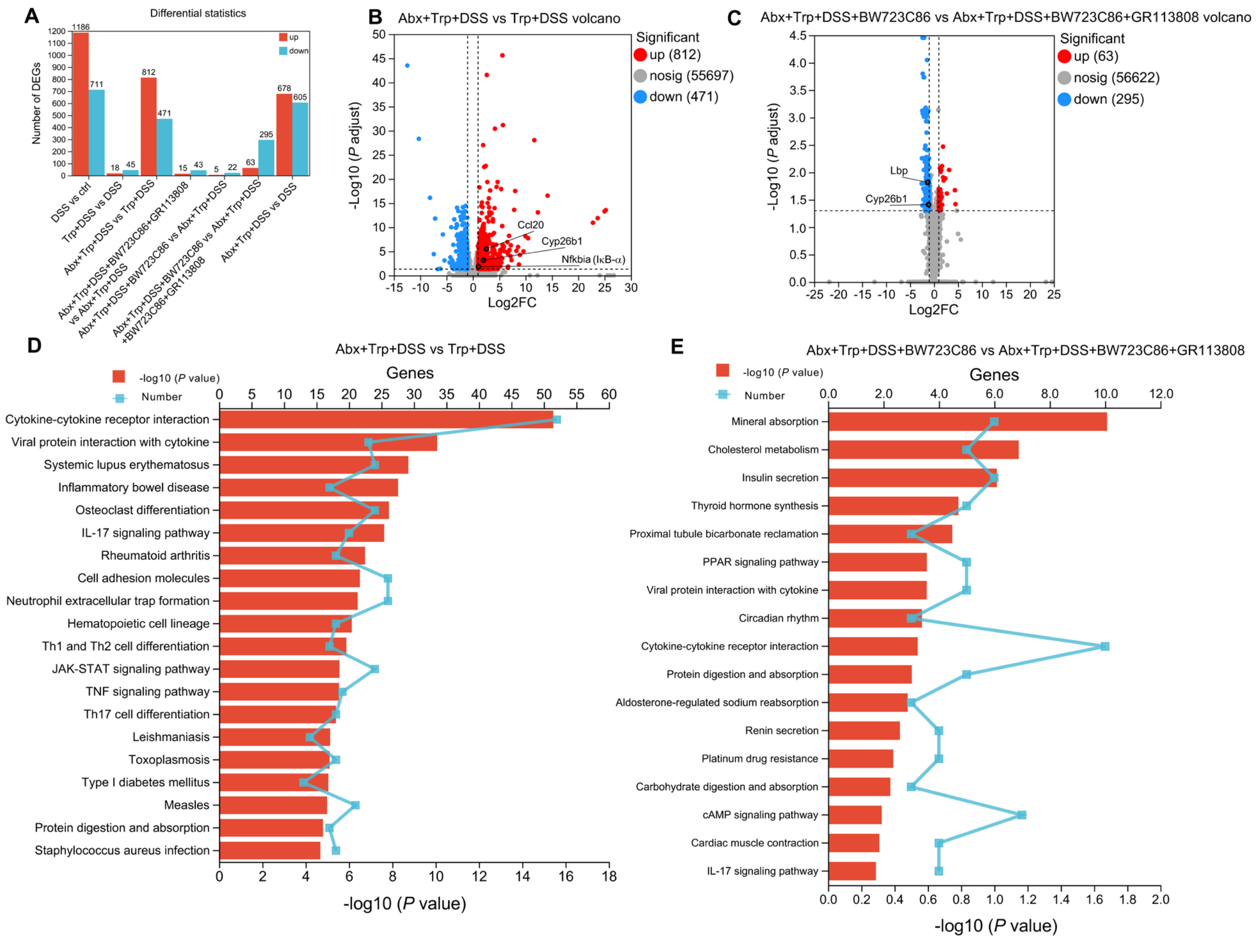

Supplementary Fig. S13 Effects of HTR2B agonist and HTR4 antagonist on transcriptional profiles in the colon of antibiotic- and Trp-treated mice with DSS-induced colitis. A Statistical histogram of differentially expressed genes (DEGs) among treatment groups. Up represents the number of upregulated DGs, while down represents the number of downregulated genes. Volcano plot of significantly differentially expressed genes between the Abx+Trp+DSS group and Trp+DSS group (**B**) and between the Abx+Trp+DSS+BW723C86 group and Abx+Trp+DSS+BW723C86+GR113808 group (**C**). Differentially enriched pathways based on KEGG analysis between the Abx+Trp+DSS group and Trp+DSS group (**D**) and between the Abx+Trp+DSS+BW723C86 group and Abx+Trp+DSS+BW723C86+GR113808 group (**E**).

Abx, antibiotic; BW723C86, HTR2B agonist; DSS, dextran sodium sulfate; GR113808, HTR4 antagonist; Trp, tryptophan.

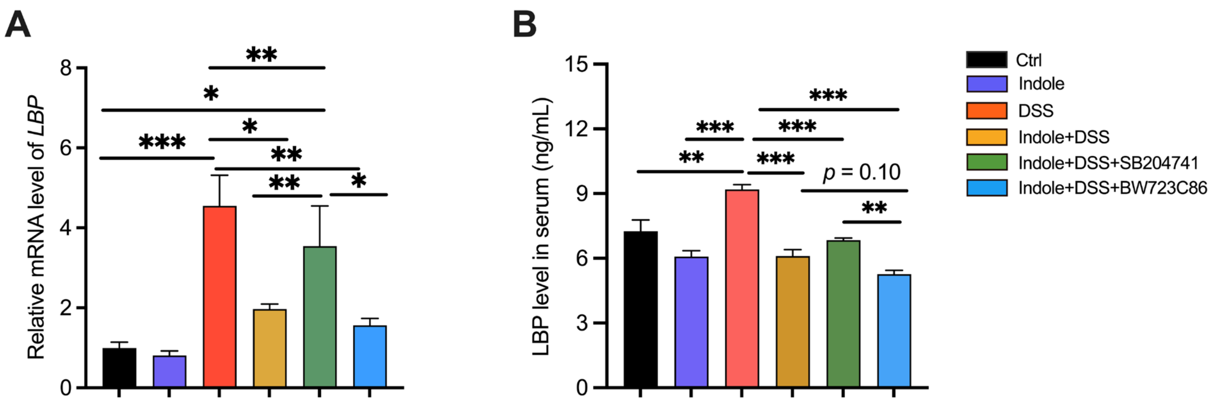

Supplementary Fig. S14 Effects of indole and HTR2B antagonist/agonist on the levels of LBP in mice with DSS-induced colitis. **A** Expression of *LBP* in the colon. **B** Levels of LBP in the serum. The data are the means ± SEMs. * $P < 0.05$, ** $P < 0.01$, *** $P < 0.001$.

Ctrl, control; DSS, dextran sodium sulfate; LBP, lipopolysaccharide-binding protein.

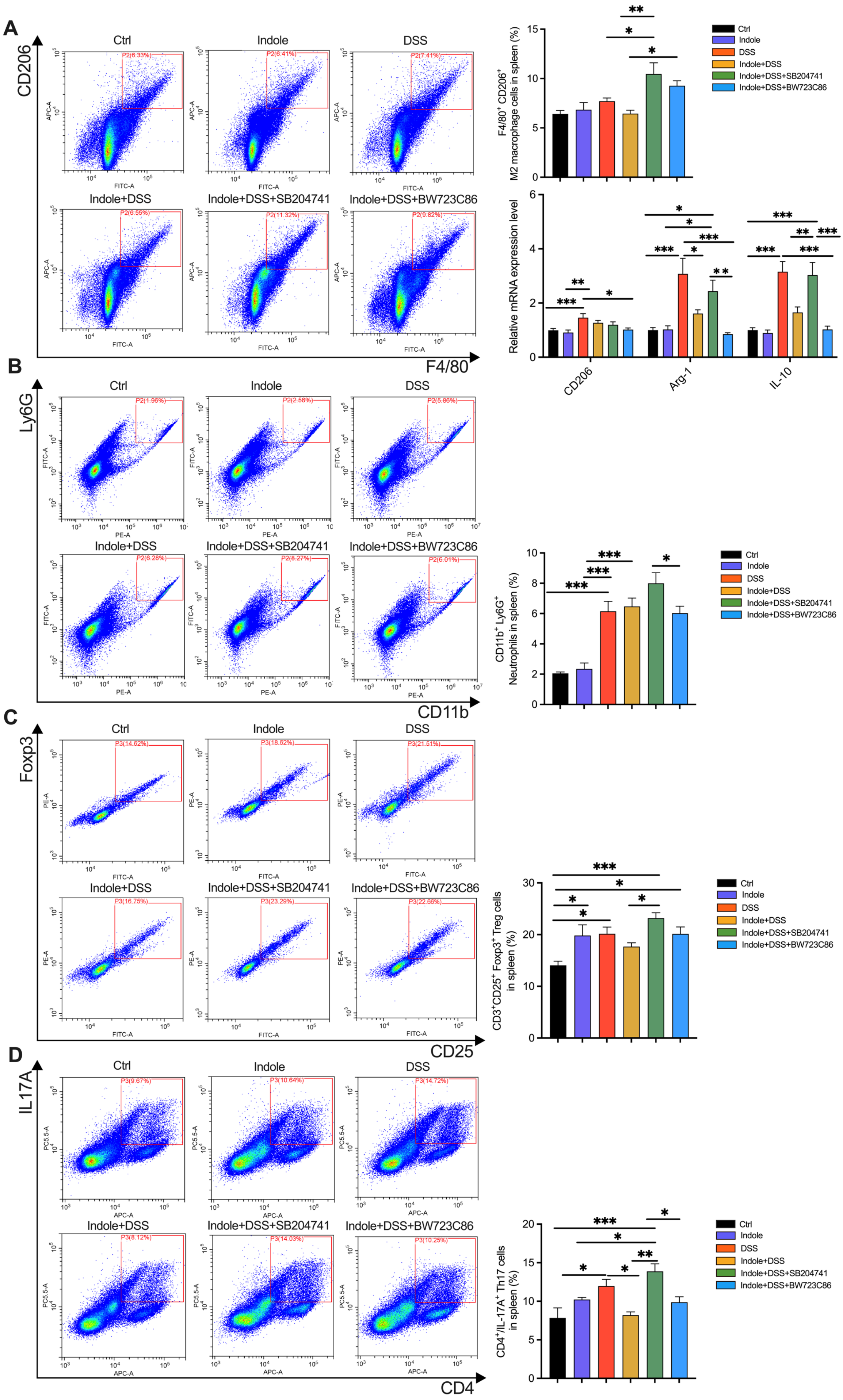
A
CD206
F4/80
Ctrl
Indole
DSS
Indole+DSS
Indole+DSS+SB204741
Indole+DSS+BW723C86
F4/80+ CD206+ M2 macrophage cells in spleen (%)
Relative mRNA expression level
CD206
Arg-1
IL-10
B
Ly6G
CD11b
CD11b+ Ly6G+ Neutrophils in spleen (%)
C
Foxp3
CD25
CD3+CD25+ Foxp3+ Treg cells in spleen (%)
D
IL17A
CD4
CD4+/IL-17A+ Th17 cells in spleen (%)

Supplementary Fig. S15 Effects of indole and HTR2B antagonist/agonist on the proportions of M2 macrophages, neutrophils, Treg cells, and Th17 cells in the spleens of mice with DSS-induced colitis. The proportions of F4/80$^+$CD206$^+$ M2 macrophages (**A**), CD11b$^+$Ly6G$^+$ neutrophils (**B**), CD3$^+$CD25$^+$Foxp3$^+$ Treg cells (**C**), and CD4$^+$IL-17A$^+$ Th17 cells (**D**) are shown. The data are the means ± SEMs. * $P < 0.05$, ** $P < 0.01$, *** $P < 0.001$.

BW723C86, HTR2B agonist; Ctrl, control; DSS, dextran sodium sulfate; SB204741, HTR2B antagonist; Th lymphocytes, T helper lymphocytes; Treg lymphocytes, regulatory T lymphocytes.

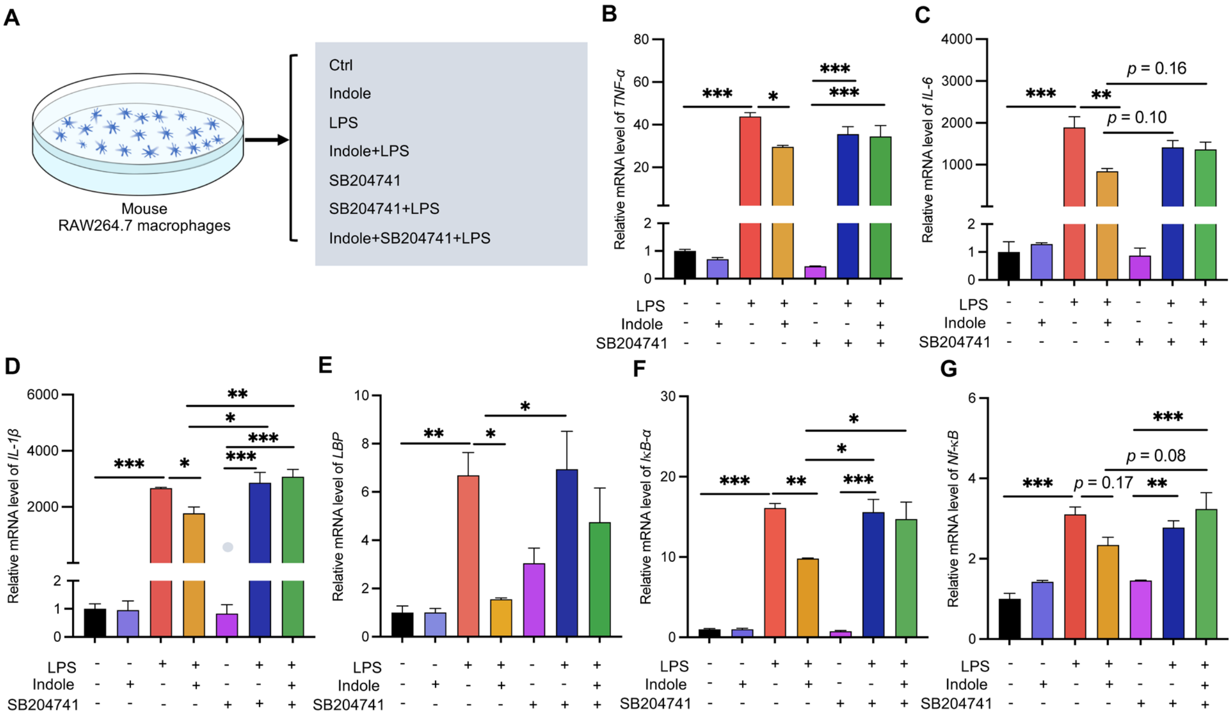

Supplementary Fig. S16 Indole suppresses the LBP-linked inflammatory response in macrophages in an HTR2B-dependent manner. A Schematic diagram of the cell culture experiment. The expression of *TNF-α* (**B**), IL-6 (**C**), IL-1β (**D**), *LBP* (**E**), *IκB-α* (**F**), and *NF-κB* (**G**). The data are the means ± SEMs. * $P < 0.05$, ** $P < 0.01$, *** $P < 0.001$.

Ctrl, control; LBP, lipopolysaccharide-binding protein; LPS, lipopolysaccharide; SB204741, HTR2B antagonist.

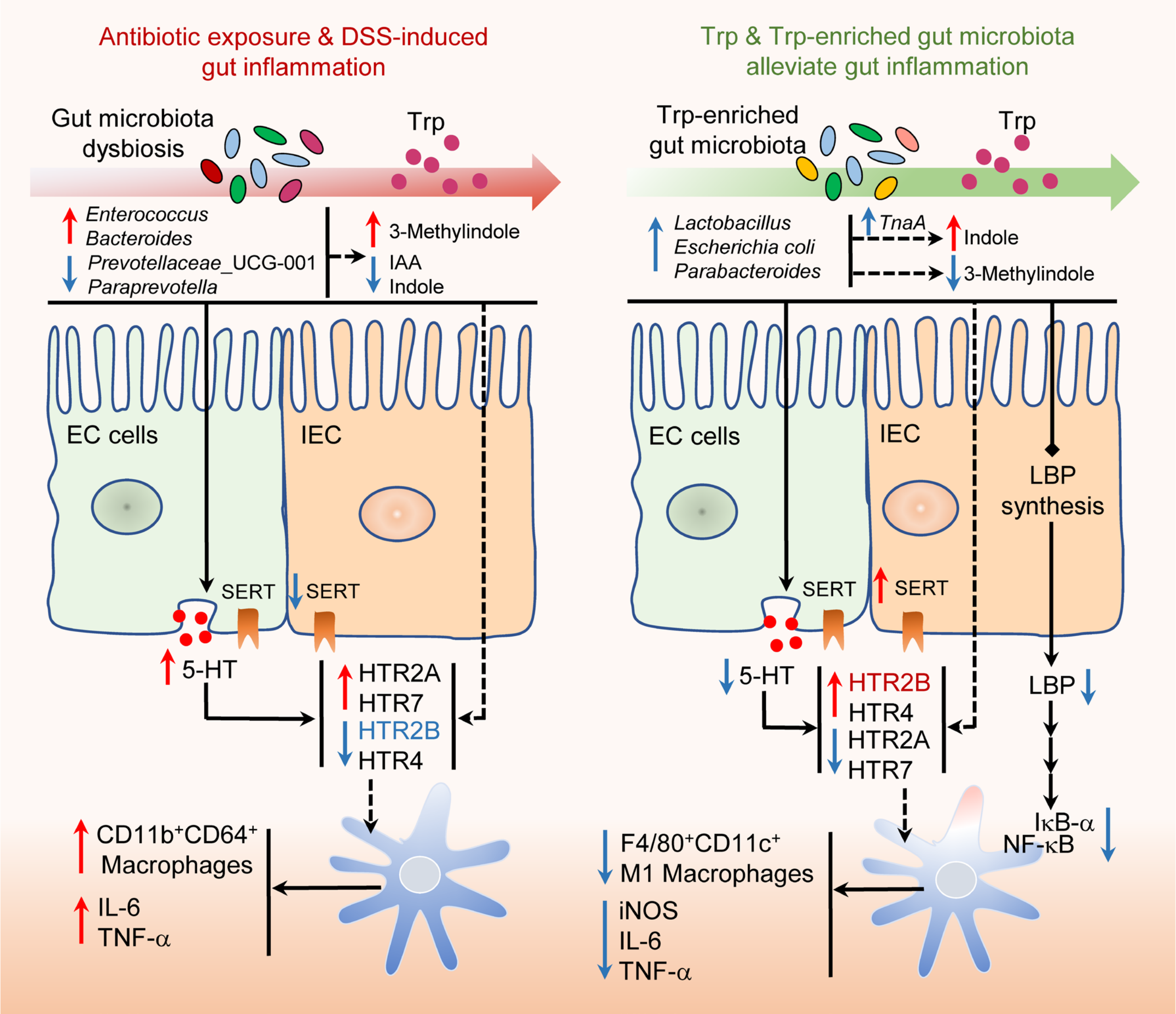

Supplementary Fig. S17 Schematic diagram of the possible mechanisms by which tryptophan (Trp) and Trp-enriched gut microbiota alleviate antibiotic exposure and dextran sulfate sodium (DSS)-induced gut microbiota dysbiosis and inflammation. The gut microbiota dysbiosis induced by antibiotic exposure before but not during DSS treatment exacerbates the immunoregulatory effects of Trp. This is due to the increased abundance of macrophages and reduced expression of the serotonin receptors HTR2B and HTR4 in the colon. Fecal microbiota transplantation of Trp-enriched microbiota from Trp-treated mice increases the levels of *Lactobacillus*, *Parabacteroides*, and indole in the colon of recipient mice pre-exposure to antibiotics. The inhibition of lipopolysaccharides-binding protein (LBP) production and IκB-α/NF-κB signaling in the colon is mediated by the activation of HTR2B and the inhibition of M1 macrophage polarization by indole.
